# Ni‐Carbon Microtube/Polytetrafluoroethylene as Flexible Electrothermal Microwave Absorbers

**DOI:** 10.1002/advs.202304218

**Published:** 2023-09-18

**Authors:** Lihong Wu, Guizhen Wang, Shaohua Shi, Xiao Liu, Jun Liu, Jinchuan Zhao, Guilong Wang

**Affiliations:** ^1^ State Key Laboratory of Marine Resource Utilization in South China Sea School of Material Science and Engineering Hainan University Haikou Hainan 570228 China; ^2^ Key Laboratory for Liquid‐Solid Structural Evolution and Processing of Materials (Ministry of Education) Shandong University Jinan Shandong 250061 China

**Keywords:** carbon materials, electrothermal effect, extreme environments, flexible film, microwave absorption

## Abstract

Flexible microwave absorbers with Joule heating performance are urgently desired to meet the demands of extreme service environments. Herein, a type of flexible composite film is constructed by homogeneously dispersing a hierarchical Ni‐carbon microtube (Ni/CMT) into a processable polytetrafluoroethylene (PTFE) matrix. The Ni/CMT are interconnected into a 3D conductive network, in which the huge interior cavity of the carbon microtube (CMT) improves impedance matching and provides additional hyper channels for electromagnetic (EM) waves dissipation, and the hierarchical magnetic Ni nanoparticles enhance the synergistic interactions between confined heterogeneous interfaces. Such an ingenious structure endows the composites with excellent electrothermal performance and improves their serviceability for application under extreme environments. Moreover, under a low fill loading of 3 wt.%, the Ni/CMT/PTFE (NCP) can achieve excellent low‐frequency microwave absorption (MA) property with a minimum reflection loss of −59.12 dB at 5.92 GHz, which covers almost the entire C‐band. Relying on their brilliant MA property, an EM sensor is designed and achieved by the resonance coupling of the patterned NCP. This work opens up a new way for the design of next‐generation microwave absorbers that meet the requirements of EM packaging, proofing water and removing ice, fire safety, and health monitoring.

## Graphical abstract

A flexible microwave absorber with excellent Joule heating performance is achieved. Based on this, an innovative patterned strain induction sensor with high sensitivity is constructed. This work opens a new gate for broadening the application and overcoming traditional constraints of MA absorbers.

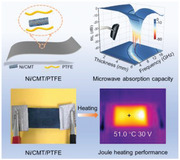

## 1 Introduction

The rapid advancement in telecommunication and the growing number of compact mobile electronic devices have inevitably caused serious electromagnetic (EM) pollution problems, including interference with communication systems, impact on human health, and potential explosion accidents.^[^ ^1^ ^−^ ^6^ ^]^ Microwave absorption materials (MAMs) as one of the effective means to tackle EM pollution are attracting more and more interest worldwide due to their ability to absorb EM wave energy and convert it into thermal energy.^[^ ^7^ ^]^ The structure design of MAMs receives wide attention because there is substantial evidence that it plays an important role in optimizing absorption strength and bandwidth. To date, multitudinous MAMs with ingenious structures have been reported to enhance microwave absorbing capacity, such as core‐shell structures,^[^ ^8^ ^]^ chiral structures,^[^ ^6^, ^9^ ^]^ porous structures,^[^ ^10^, ^11^ ^]^ and hierarchical architectures.^[^ ^12^, ^13^ ^]^ However, numerous studies currently focused on improving EM absorption abilities of MAMs.^[^ ^9^, ^14^, ^15^ ^]^ Rare effort has been devoted to strengthening their environmental suitability. In essence, the real resistance for absorbers to engineering applications is the harshness and complexity of the practical application environment. For example, electronic devices or equipment might cause failure or their performance might be decreased when working in humid conditions. Besides, the surface icing in extremely cold conditions would also invalidate the microwave absorption (MA) performance of MAMs. Therefore, developing a highly tolerant microwave absorber to address these issues is urgently desirable.

Surface coatings with electrothermal function as one of the most attractive strategies were employed frequently to displace moisture and deicing in various fields.^[^ ^16^, ^17^ ^]^ By utilizing the Joule heating effect, electronic devices can be maintained at a constant temperature due to sustainable heat release.^[^ ^17^ ^]^ Therefore, the innovative design of high‐efficiency microwave absorbers with good Joule heating performance may be a promising solution to meet the severe requirements of MAMs under extreme environments. To better combine MA property with electrothermal function, carbon materials stand first of all candidates owing to their excellent thermal and electrical conductivity, light weight, good dielectric loss, and high thermal stability.^[^ ^13^, ^18^, ^19^ ^]^ Nevertheless, for pure carbon materials, the high electrical conductivity usually leads to excessive conductive loss, which tends to cause a large amount of incident EM wave to be reflected rather than absorbed.^[^ ^20^, ^21^ ^]^ Simultaneously, the unsatisfied impedance matching and simplex loss mechanism also hinder their EM wave attenuation ability.^[^ ^22^ ^]^ Furthermore, the strong large interfacial thermal resistance and poor dispersion between matrix and pure carbon material limit their function for electrothermal performance.^[^ ^11^ ^]^ Therefore, further optimizing both the MA property and the electrothermal performance of carbon materials is urgently necessary.

Here, a well‐designed MA film with electrothermal function and good flexibility is successfully fabricated by employing the hierarchical Ni‐carbon microtube (Ni/CMT) with both dielectric and magnetic features as filler and polytetrafluoroethylene (PTFE) with good chemical corrosion resistance and flame resistance as a flexible matrix. Ni nanoparticles uniformly distributed in the outer and inner of carbon microtube (CMT) by the pyrolysis of Ni metal‐organic framework (Ni‐MOF) can form multi‐interface configurations and 3D magnetic coupling networks. The synergistic effect of heterogeneous interfaces can induce strong interfacial polarization, and the huge interior cavity can improve impedance matching and produce multiple reflections. Moreover, an appropriate buffer layer of magnetic Ni nanoparticles uniformly dispersed on the CMT as the harmonic medium facilitates the modulus matching, which ameliorates the interfacial thermal resistance. Furthermore, the encapsulation of the PTFE polymer network not only enhances Ni/CMT adhesion, thereby preventing Ni nanoparticles shedding and oxidation, but also improves the composite anticorrosion and stability. Consequently, the Ni/CMT/PTFE (NCP) film achieves low‐frequency MA with a minimum reflection loss (RL_min_) of −59.12 dB at 5.92 GHz, and the effective absorption bandwidth (EAB) covers almost the entire C‐band at a loading of only 3 wt.% Ni/CMT, surpassing the performance of most reported absorbers. In particular, the NCP film also exhibits excellent Joule heating ability, hydrophobicity, and fire‐retardant properties. Based on these, an innovative patterned strain induction sensor with high sensitivity is constructed. Such research opens a new gate for broadening the application and overcoming traditional constraints of MA absorbers.

## 2 Results and Discussion

### 2.1 Effect of Morphology and Structure on EM Properties

**Figure 1:**
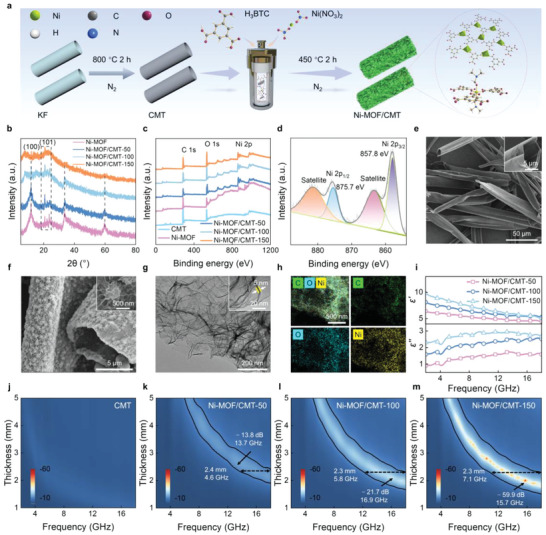
Fabrication, structural characterization, and EM properties of Ni‐MOF/CMT. a) Schematic illustration of fabricating hierarchical Ni‐MOF/CMT. b) XRD patterns and c) XPS spectra of Ni‐MOF, Ni‐MOF/CMT‐50, Ni‐MOF/CMT‐100, and Ni‐MOF/CMT‐150. d) XPS spectrum of Ni 2p. SEM images of e) CMT and f) Ni‐MOF/CMT‐150. g) TEM image and h) EDS elemental mappings of Ni‐MOF/CMT‐150. i) *ε′* and *ε″* values of Ni‐MOF/CMT samples. Reflection loss (RL) curves of j) CMT, k) Ni‐MOF/CMT‐50, l) Ni‐MOF/CMT‐100, and m) Ni‐MOF/CMT‐150, respectively.

Above all, the structure of the Ni‐MOF/CMT was characterized to analyze the effect of chemical composition and microstructure on EM properties, respectively. **Figure** **1**a schematically illustrates the design strategy of Ni‐MOF/CMT hierarchical microtubes. Kapok fiber (KF) was selected as the template of CMT because of its natural microtubular architecture, large lumens, thin walls, high aspect ratio, and low density. Ni‐MOF/CMT was prepared via a facile hydrothermal method of assembling the Ni^2+^ with trimesic acid (H_3_BTC), with tunable morphology and typical crystalline structure. The process details are outlined in the Supporting Information. To explore the impact of the CMT amount on the microstructure evolution of Ni‐MOF/CMT, the samples with 50, 100, and 150 mg of CMT were synthesized and referred to Ni‐MOF/CMT‐50, −100, and −150, respectively. X‐ray diffraction (XRD) patterns of the Ni‐MOF and the Ni‐MOF/CMT are presented in Figure 1b. The high‐intensity peaks at ≈10^o^ and 22° correspond to (100) and (101) planes, respectively, which are characteristic peaks of Ni‐MOF coordinated by Ni^2+^ and H_3_BTC.^[^ ^23^, ^24^ ^]^ X‐ray photoelectron spectroscopy (XPS) spectra of Ni‐MOF/CMT show the presence of C, O, and Ni elements (Figure 1c,d). In Ni 2p spectrum, two main peaks at 857.8 and 875.7 eV can be assigned to Ni 2p_3/2_ and Ni 2p_1/2_ spin orbits, respectively.^[^ ^25^ ^]^ At the same time, two peaks located at ≈863.3 and 881.5 eV are shake‐up satellites of Ni 2p_3/2_ and Ni 2p_1/2_, respectively, confirming the existence of Ni^2+^.^[^ ^24^ ^]^

Typical scanning electron microscopy (SEM) images of KF display a tubular structure with diameters in the range of 10−40 µm (Figure S1a,b, Supporting Information). In Figure 1e and Figure S1c (Supporting Information), the CMT inherits the overall morphology of KF with a huge interior cavity, morphological uniformity, and smooth surface. Energy‐dispersive spectroscopy (EDS) elemental mapping of CMT reveals the homogeneous distributions of C, N, and O elements (Figure S1d, Supporting Information), which is beneficial for increasing defects and improving electrical conductivity. A hydrothermal reaction was employed to synthesize hierarchical microtubes. It is clear that the ultrathin and uniform Ni‐MOF nanoflakes are uniformly anchored on both the outer and inner surfaces of CMT, and they intersect each other. Consequently, the hierarchical microtubes with hetero‐interfaces and hollow cavities are obtained (Figure 1f; Figures S1 and S2, Supporting Information). The formation of the hierarchical architecture can be explained as follows: Ni^2+^ can be absorbed by CMT via electrostatic attraction and generate nucleation centers to assist the crystallization and growth of Ni‐MOF.^[^ ^24^, ^26^ ^]^ Subsequently, the deprotonated H_3_BTC ligands are coordinated with Ni^2+^, and the bridged ordered chains self‐assemble into a 3D porous array structure along with the CMT framework. In addition, the effect of CMT amount on the morphology of Ni‐MOF/CMT was also investigated (Figure S2, Supporting Information). In the absence of CMT, Ni‐MOFs freely nucleate and grow, resulting in layer‐by‐layer stacked structures consisting of aggregated 2D nanoflakes (Figure S3, Supporting Information). For small amounts of CMT, the aggregated nanoflakes adhered to the CMT are observed, leading to reduced void spaces. With the increase of CMT amount, the loose and uniform morphology is obtained, which may be ascribed to the uniform distribution in nucleation sites. Therefore, the morphology of Ni‐MOF/CMT can be accurately controlled by adjusting the amount of CMT. In this strategy, the moderate CMT can effectively tune the aggregation degree of Ni‐MOF, thus forming a loose sheetlike structure. This unique 3D structure can result in extra void space and large surface area, which is in favor of EM wave scattering. The transmission electron microscopy (TEM) image further confirms that the Ni‐MOF nanoflakes adhered to the CMT surface compactly with random orientation and the thickness of nanoflakes are ≈5 nm (Figure 1g). These results are also supported by high‐angle annular dark field‐scanning TEM (HAADF‐STEM) and EDS elemental mappings (Figure 1h; Figure S4, Supporting Information), which is consistent with XPS data. This further indicates that Ni‐MOF nanoflakes are uniformly distributed on the CMT and form a homogeneous micro‐nanostructure.

Generally, the ability of MA is determined by EM parameters (Equations S1 and S2, Supporting Information).^[^ ^27^, ^28^ ^]^ The real permittivity *ε'* (permeability *µ′*) and imaginary permittivity *ε″* (permeability *µ′′*) represent the capacity to store and attenuate dielectric (magnetic) energy (Equations S3 and S4, Supporting Information).^[^ ^29^ ^]^ Although MOF materials exhibit outstanding physical‐chemical characteristics, unique porous structure, and significant specific surface area^[^ ^15^, ^30^ ^]^ which offer great advantages for MA, the low dielectric constant limits their ability to efficiently absorb microwave energy. The as‐synthesized Ni‐MOF reveals almost constant *ε′* and *ε″* values of 2.2 and 0.1, respectively, implying the penetration of most incident microwave through the whole Ni‐MOF at the measurement frequency. By contrast, the pristine CMT shows a high *ε′* and *ε″* range from 48.7 to 12.5 and 34.4 to 16.7, respectively (Figure S5, Supporting Information). This indicates that the magnetic and dielectric properties are mismatched in this case, reducing the effective absorption of EM wave. Introducing Ni‐MOF into CMT is an effective strategy to optimize impedance matching and thus improve its MA performance (Figure S6, Supporting Information). After growing with Ni‐MOF, the *ε*′ and *ε*″ values of Ni‐MOF/CMT rapidly decrease with the increasing Ni‐MOF amount (Figure 1i). With increasing frequency, the *ε*′ values of Ni‐MOF/CMT‐50, Ni‐MOF/CMT‐100, and Ni‐MOF/CMT‐150 decline from 6.4 to 4.5, 8.1 to 5.3, and 9.8 to 5.1, respectively. Simultaneously, the *ε*″ values of Ni‐MOF/CMT‐50, Ni‐MOF/CMT‐100, and Ni‐MOF/CMT‐150 increase from 0.9 to 1.6, 1.6 to 2.5, and 2.3 to 2.7. Such improvement for Ni‐MOF/CMT‐150 can be attributed to the fact that CMT effectively modulated the layer thickness of Ni‐MOF and enhanced the conductivity of integrated architecture, thus resulting in high conductive loss, optimized impedance matching, and enough numerous defects and surface functional groups as dipolar polarization sites. In this way, compared with pure CMT and Ni‐MOF, the MA capability of Ni‐MOF/CMT is dramatically enhanced. As shown in Figure 1j–m and Figure S7 (Supporting Information), the Ni‐MOF/CMT‐50 shows a low level of MA performance with a RL_min_ value of −13.8 dB and a maximum EAB of 4.6 GHz. With an increase in the CMT content, Ni‐MOF/CMT‐100 shows a broadband MA ranging from 12.2 to 18.0 GHz at the thickness of 2.3 mm and the RL_min_ can achieve −21.7 dB at 16.9 GHz. Comparatively, the Ni‐MOF/CMT‐150 with optimal CMT content presents the best MA property accompanied by an excellent wideband of 7.1 GHz in the range of 10.9 to 18.0 GHz at 2.3 mm, and a RL_min_ of −59.9 dB at 2.0 mm. The excellent MA capacity of the Ni‐MOF/CMT suggests that the dielectric performance of Ni‐MOF can be effectively enhanced by introducing CMT, and perfectly preserving the structure of Ni‐MOF is advantageous for MA.

### 2.2 Morphology and Structure of NCP

**Figure 2:**
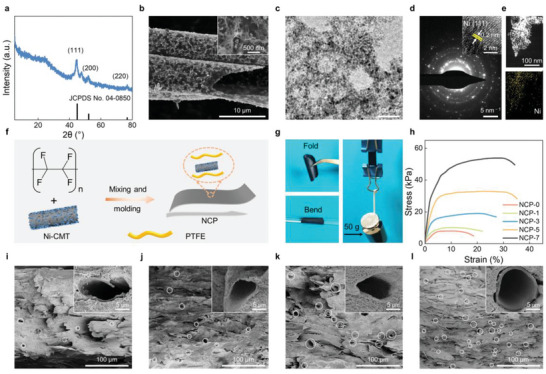
Identification of Ni/CMT and structural characteristics of NCP films. a) XRD pattern, b) SEM image, c) TEM image, and d) SAED image (inset is HRTEM image) of Ni/CMT. e) HAADF‐STEM image and corresponding EDS elemental mapping of Ni/CMT. f) Schematic illustration for preparing NCP films. g) Digital photos of NCP‐7. h) Tensile stress−strain curves. SEM cross‐sectional images of i) NCP‐1, j) NCP‐3, k) NCP‐5, and l) NCP‐7.

Microwave absorbers with high tolerance and stability have a better application prospect in harshness and complex service environments. Herein, a high‐temperature treatment was performed on Ni‐MOF/CMT‐150 to prepare the stable Ni/CMT. The structure and morphology characters of Ni/CMT are shown in **Figure** **2**a–d. The high‐temperature annealing contributed to the construction of thermodynamically stable cubic Ni (JCPDS No. 04–0850) (Figure 2a).^[^ ^31^ ^]^ The morphology of Ni‐MOF/CMT after annealing is well maintained, but the size of the nanoflake is shrinking due to the loss of organic ligands at high temperatures (Figure 2b). Apparently, the hierarchical Ni are constructed by nanoparticles with a size of ≈5−20 nm (Figure 2c). The polycrystalline structure of Ni/CMT can be confirmed by the selected area electron diffraction (SAED) pattern (Figure 2d). The high‐resolution (HR) TEM image shows the interplanar spacing of the (111) crystal plane of Ni/CMT (0.2 nm) (inset in Figure 2c),^[^ ^31^, ^32^ ^]^ which is in accordance with XRD data. EDS mapping image and XPS spectrum further reveal the existence of Ni in Ni/CMT (Figure 2d; Figure S8, Supporting Information).

Conventional MAMs are applied as coatings on the protected targets to absorb microwaves, the weak adhesion of absorbers and targets greatly limits its application for MA. Ni/CMT can be fixed by PTFE in the frame structure to prepare a flexible NCP‐*x* (*x* presents the percentage of Ni/CMT) film (Figure 2f). As we expected, the composite films exhibit remarkable flexibility and excellent mechanical properties. They can be folded in half, bent, and withstand a weight of 50 g without any wrinkles. (Figure 2g). After linear elastic deformation and yielding, the composite films exhibit obvious plastic elongation and eventually fracture (Figure 2h). The outstanding mechanical flexibility of the NCP films enables them to conform to various complex shapes without shedding. The SEM images of NCP films exhibit a dense surface morphology without visible cracks or defects (Figure S9, Supporting Information). Moreover, Ni/CMT is positioned in the PTFE in the form of high‐integrity hollow microstructures with a homogenous distribution (Figure 2i–l). As the Ni/CMT content increases, multiple Ni/CMT is stacked on top of each other to form a 3D conductive network structure, which is conducive to the dissipation of EM wave to enhance the MA performance.

### 2.3 Electrothermal Properties and Fire Safety of NCP Films

**Figure 3:**
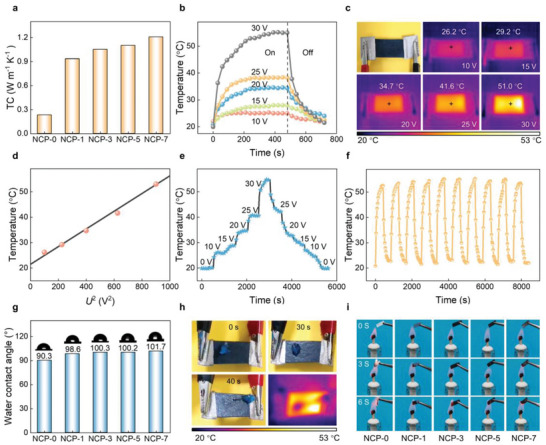
Joule heating, hydrophobic, and fire resistance properties of NCP. a) TC of NCP samples. b) The surface temperature of NCP‐7 with various driven voltages. c) The photograph and thermal infrared images for NCP‐7 at different driven voltages. d) Experimental data and linear fitting of saturation temperature for NCP‐7 versus *U* ^2^. e) The tailored surface temperature of NCP‐7 under voltage gradient variation. f) The surface temperature−time curve of NCP‐7 during the cyclic on−off tests at 30 V. g) Water contact angles of NCP films. h) Digital photos and thermal infrared image for NCP‐7 at a working voltage of 30 V during the deicing process. i) Fire retardance test of NCP films.

Expectantly, the Joule heating performance of the NCP films was systematically investigated. NCP‐7 was chosen as the representative for the following elaboration. The high electrical and thermal conductivity of NCP film is necessary for the Joule heater. The intercalating Ni/CMT as an electrical conductor into the NCP film forms the conductive path and the increased content results in a continuous increase in conductivity. The increase of conductivity further confirms the formation of a 3D conductive network. For NCP‐7, its electrical conductivity is 0.93 S m^−1^ (Figure S10, Supporting Information), which helps to generate Joule heating with weak electrical signals. Moreover, the thermal conductivity (TC) of the NCP films gradually increases with the increasing Ni/CMT content, and at 7 wt.% Ni/CMT content, it reaches 1.2 W m^−1^ K^−1^ (**Figure** **3**a). Figure 3b shows the surface temperature of NCP‐7 as a function of time under a low voltage of 10−30 V. At 10 V, the film can produce a comfortable temperature of 26.2 °C. A plateau is observed in the heating and cooling processes, indicating that the NCP‐7 exhibits a good heat storage effect and can continue to provide energy for the microwave absorber after fully absorbing thermal energy. The change of saturated temperature with driving voltages is visually shown by infrared thermal images (Figure 3c). The square of applying voltage (*U* ^2^) displays a good linear relationship with the saturated temperature (Figure 3d), which is consistent with the theoretical prediction. It should be noted that the surface temperature of NCP‐7 can be rapidly switched when the driven voltage is adjusted (Figure 3e). In addition, the surface temperature of NCP‐7 remains stable during 10‐cycle one‐off tests at 30 V (Figure 3f), testifying to its reliability and durability of power‐driven heat conversion. All of the above results indicate the good Joule heating recyclability, controllability, and stability of the NCP‐7 for heat management applications. The NCP films exhibit hydrophobicity, as evidenced in Figure 3g, in which the water contact angle of NCP‐7 can reach 101.7°. Besides, the synergistic effect of Joule heating performance and hydrophobicity can realize a desirable deicing effect. As shown in Figure 3h, blue ice is placed on a sloping NCP‐7 heater at a driven voltage of 30 V. The ice melts and slips away from the film surface within 40 s, which is important for microwave absorber applications under extreme experiments.

Fire safety is a crucial parameter for MA performance and thermal management. Inevitably, a fire hazard may be posted when the electronic equipment is operating at high speed. Hence, it is important to construct a barrier of a fire‐resistant film to control the spread of fire. To investigate the fire safety of NCP films, the alcohol lamp burning experiments were characterized (Figure 3i). After burning for 3 s, NCP‐0 burns quickly and droplets appear during the combustion process, while NCP‐1 exhibits self‐extinguishing and its shape is maintained during the entire burning process. Moreover, NCP‐3, NCP‐5, and NCP‐7 also show good fire resistance. Meanwhile, a slight curling of NCP‐7 is observed after combustion, suggesting the threshold of Ni/CMT content in the fire resistance effect. In a limiting oxygen index (LOI) test (Table S1, Supporting Information), a slight increase in LOI values is observed with Ni/CMT content from 1 to 7 wt.%. The fire resistance performance of NCP films can be attributed to the physical barrier of Ni/CMT, Ni/CMT hybrid protective layer not only hinders the diffusion of oxygen into PTFE and the migration of volatile decomposition products out of PTFE but also acts as a thermal shield for energy feedback from the flame.^[^ ^17^ ^]^

### 2.4 Efficient MA Performance

**Figure 4:**
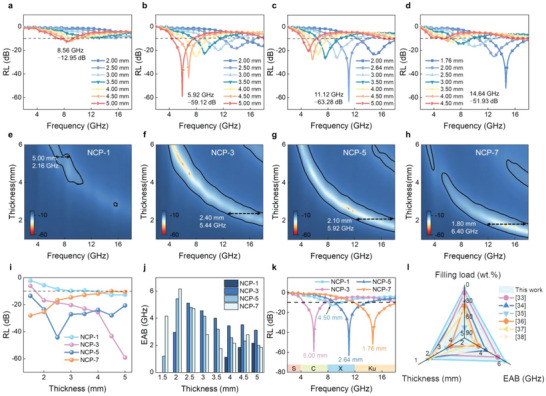
MA performance of NCP samples. RL curves of a,e) NCP‐1, b,f) NCP‐3, c,g) NCP‐5, and d,h) NCP‐7. i) RL values and j) EAB values of NCP samples under different thicknesses. k) Optimal RL plots in the C‐band (4−8 GHz), X‐band (8−12 GHz), and Ku‐band (12−18 GHz) of the series NCP. l) Comparison of the reported Ni‐based absorbers.

The NCP films also exhibit highly efficient MA performance in the frequency of 2−18 GHz (**Figure** **4**; Figure S11, Supporting Information). The RL_min_ of NCP‐0 is merely −3.45 dB due to the poor dielectric loss ability and the value increases to −12.95 dB for NCP‐1 (Figure 4a,e; Figure S12, Supporting Information). The NCP‐3 exhibits a low‐frequency MA, and the RL_min_ reaches −59.12 dB at 5.92 GHz when the thickness is 5.00 mm (Figure 4b), which covers almost C‐band. Moreover, an EAB of 5.44 GHz at 2.40 mm thickness is obtained (Figure 4f). With an increase in the Ni/CMT content, NCP‐5 shows a broadband MA ranging from 11.92 to 17.84 GHz at the thickness of 2.10 mm and the RL_min_ can achieve −63.28 dB at 11.12 GHz (Figure 4c,g). Particularly, at an ultra‐thin thickness, NCP‐7 presents the best MA property accompanied by an excellent wideband of 6.40 GHz at 1.80 mm and a RL_min_ of −51.93 dB at 1.76 mm (Figure 4d,h), confirming its great strong potential for MA and related applications. Figure 4i,j plots the RL and EAB for NCP samples at the thickness of 1.5−5.0 mm. Interestingly, all NCP samples display an effective absorption with a moderate bandwidth except for NCP‐1 with a very low content of Ni/CMT, indicating that they are ideal candidates for lightweight absorbers. Notably, by tailoring the content of Ni/CMT from 1 to 7 wt.%, the EAB of NCP composites can cover all of Ku‐band, X‐band, and C‐band (4.4 to 18.0 GHz) (Figure 4k). Such superior MA performance at ultra‐low fill loadings is rarely reported in previous work (Figure 4l).^[^ ^33^ ^−^ ^38^ ^]^

**Figure 5:**
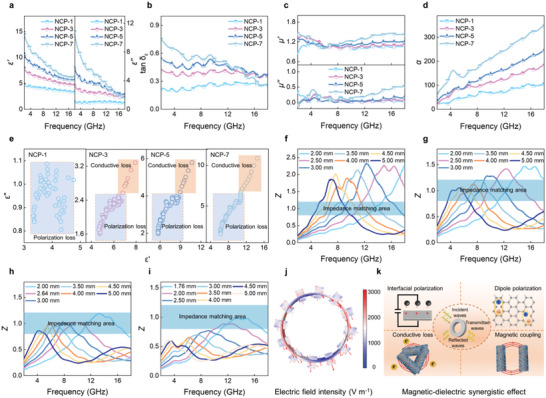
MA mechanism of NCP samples. a) *ε*′ and *ε*″, b) tan δ*_ε_*, c) *µ*′ and *µ*″, d) α, and e) Cole‐Cole plots of NCP samples. Z curves of f) NCP‐1, g) NCP‐3, h) NCP‐5, and i) NCP‐7. j) The electric field polarization direction and the distribution of the electric field norm. k) Schematic illustration of MA mechanisms for NCP.

To investigate the associated mechanism, the EM parameters of as‐synthesized samples are plotted in **Figure** **5** and Figure S13 (Supporting Information). The NCP‐1 shows the lowest complex permittivity due to the inferior conductivity at the low Ni/CMT content (Figure 5a). With increasing the Ni/CMT content, the 3D conducting network is emerging, resulting in higher conductivity and increased complex permittivity. The dielectric loss tangent (tan δ*_ε_* = *ε′′/ε′*) also shows a similar trend with the *ε*′′ (Figure 5b). The complex permeability of all NCP samples shows almost identical values without any noticeable decay in the entire microwave frequency (Figure 5c). Attenuation constant (*α*) is a vital factor for evaluating the MA performance of the absorber, which determines the ability of EM wave energy to be converted into other forms of energy (Equation S5, Supporting Information).^[^ ^27^ ^]^ Here, the *α* values of all samples increase with the improving Ni/CMT content and increase with the increasing frequency (Figure 5d). In addition, Cole‐Cole plots reveal the polarization loss (Equation S6, Supporting Information), as plotted in Figure 5e. The slender tail at the rightward area represents conductive loss and the leftward semicircles area is associated with the polarization relaxation process.^[^ ^39^, ^40^ ^]^ The semicircles are observed in all samples, indicating the multiple polarization relaxation processes. Consistent with the electric conductivity data (Figure S10a, Supporting Information), the line tails of samples increase with the addition Ni/CMT. The absence of the line tail for NCP‐1 is due to poor conductivity. Polarization loss is produced by the deflection of the dipoles with the direction of the external EM field. The dipole rearrangement cannot catch up with changes in the EM field, resulting in dielectric relaxation losses.^[^ ^27^ ^]^ Numerous functional groups or defects on CMT act as dipole active sites. Their asymmetric distribution of charges contributes to intensive dipole polarization. The charge accumulation between CMT and Ni ensures strong interfacial polarization, which further facilitates the improvement of MA property. Moreover, the void spaces introduced by hierarchical Ni nanoparticles facilitate the multiple reflections and scattering that extend the propagation path of EM wave and are helpful to the loss of EM energy. The related mechanisms are summarized in Figure 5k. Impedance matching (*Z*) is a crucial factor that should be considered in the design of the absorbers, and the *Z* value close to 1 is a prerequisite for good MA performance.^[^ ^41^ ^]^ The *Z* values of NCP‐1 are >1.5, and the *Z* values are significantly optimized with the increase of Ni/CMT content (Figure 5f–i). Particularly, NCP‐7 exhibits the best impedance matching areas in the range of 0.8−1.2 at thin thickness. The *Z* contour maps of the samples intuitively depict the impedance matching property (Figure S14, Supporting Information). All these results indicate that absorbers with excellent MA performance not only need large attenuation capabilities to meet energy losses but also meet impedance matching. Additionally, the peak frequency (*f*_m_) of all samples shifts toward the low‐frequency region with increasing matching thickness (*t*_m_) (Figure S15, Supporting Information). The pentagram signifying the experimental *t*_m_ agrees well with the simulated line, which was plotted by the quarter‐wavelength (λ/4) matching theory (Equation S7, Supporting Information).^[^ ^42^ ^]^ This indicates that the strong MA of NCP samples satisfies the λ/4 matching theory, which suggests an interference‐type loss.

To deeply understand the MA mechanism of NCP, the electric field polarization direction, electric field intensity, and electric energy loss distribution at the frequency of 14.6 GHz were simulated by COMSOL Multiphysics (Figure 5j; Figure S16, Supporting Information). The electric field polarization direction is changed, indicating that EM wave pass through the absorber, and can be repeatedly reflected and scattered on the hierarchical interfaces. When an EM wave incident into NCP, the excited electrons in CMT migrate along the axis or jump to the neighboring Ni through interfaces and defects, which is vital for the electric energy loss. In addition, the formation of gradient impedance after introducing the Ni into CMT is conducive to more EM wave propagating into the absorber and being repeatedly reflected and scattered on the hierarchical interfaces, which leads to more energy dissipated since the transmission path of EM wave is extended. This indicates that the unique hierarchical tubular structure of Ni/CMT endows NCP with an excellent MA performance.

**Figure 6:**
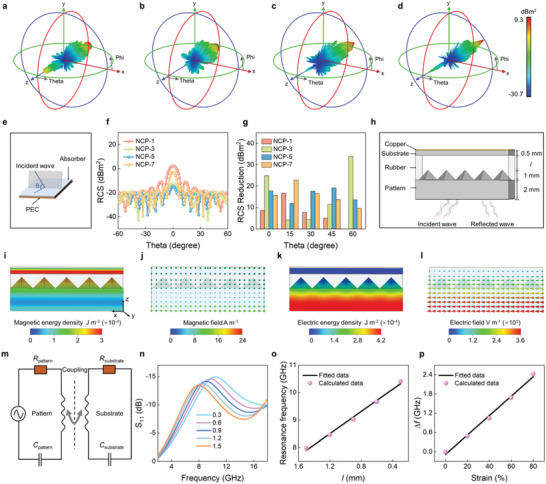
RSC simulation results and pressure‐driven EM sensor. 3D RCS diagram for the PEC substrate covered with a) PCN‐1, b) PCN‐3, c) PCN‐5, and d) PCN‐7. e) RCS simulation model. f) RCS curves and g) RCS reduction values of NCP composites. h) Schematic illustration of the sensor (0.1 mm thickness of copper). i) Magnetic energy density distributions, j) magnetic field, k) electric energy density distributions, l) electric field of NCP‐7 pattern at l of 0.3 mm. m) Equivalent circuit model. n) Frequency‐dependence *S*_11_ at *l* = 0.3−1.5 mm. o) Resonance frequency response fitted curve with *r* ^2^ = 0.9926 at various *l*. p) Fitted pressure‐response curve with *r* ^2^ = 0.9925 (pressures are represented by the *l* of rubber).

To evaluate the actual far‐field condition MA capacities in real situations, a radar cross‐section (RCS) simulation was carried out of NCP absorbers (**Figure** **6**a–g; Figure S17, Supporting Information). In the simulation model, the positive z‐axis is chosen as the direction of EM waves incidence and theta is the detection angle.^[^ ^43^ ^−^ ^45^ ^]^ The scattering signal intensity can be reflected by radiation lobe structure and color. The lower the intensity of the scattered signal, the stronger the absorption capacity. To investigate the RCS simulation at the strongest absorption frequency, the 3D radar wave scattering signals of the perfect electric conductor (PEC) layer and the PEC layer covered NCP samples were obtained. Compared with the PEC plate, the four plates coated with NCP samples show lower scattering (Figure 6a–d; Figure S17, Supporting Information). Especially, the scattering signal of the plate coated with NCP‐1 is higher than those of the other NCP systems, which is consistent with the best RL property of NCP‐1 among NCP systems (Figure 4). Moreover, 2D RSC values are plotted in Figure 6f. When EM waves incident perpendicularly to the model plane, the EM waves cover a larger proportion. The RCS values gradually decrease from 0° to −60° and 60° with several fluctuations. The RSC value of NCP‐7 is < −10 dBm^2^ over the range of −60° < theta < −5° and 5° < theta < 60°, while the RSC value of NCP‐3 and NCP‐5 are < −10 dBm^2^ over the entire detection angle, indicating the promising radar wave absorption candidate of NCP. To gain further insight, the RCS reduction values (the RCS values of PEC subtract that of the specimens) are depicted in Figure 6g. All samples make contributions to reducing RCS. In particular, NCP‐1 contributes 16.7 dBm^2^ of RCS reduction at 15°, NCP‐3 contributes 34.1 dBm^2^ of RCS reduction at 60°, NCP‐5 contributes 19.2 dBm^2^ of RCS reduction at 45°, and NCP‐7 contributes 22.9 dBm^2^ of RCS reduction at 15°. The RCS simulation results indicate the fabulous radar wave attenuation capacity, which can suppress the scattering and reflection of microwaves from the surface of PEC.

### 2.5 EM Sensor

The NCP composites were chosen to design a pattered strain sensor with a pressure recording function. A rubber with wave‐transparent and high elasticity properties is sandwiched by a pattern of NCP Figure 6h.^[^ ^46^ ^−^ ^48^ ^]^ The energy density distributions present the magnetic loss mainly concentrated in the substrate layer and electric loss occurred in the patterned layer at *f* = 9 GHz (Figure 6i,k; Figure S18, Supporting Information). Figure 6j,l shows the magnetic vectors resonate along the y‐axis and the electric vectors resonate along the x‐axis. The sensor can be equivalent to a circuit that consists of the inductance, capacitance (C), and resistance (R) of each component (Figure 6m).^[^ ^46^ ^]^ The sensor is sensitive to pressure that can be characterized by the strain of rubber. When compressing the rubber height (*l*) from 1.5 mm to 0.3 mm, the coupling effect between the two NCP layers increases, the *S*_11_ peak shifts blue (Figure 6n), and the resonance frequency shift about 2.4 GHz (Figure 6o). A linear increment rate of resonance frequency (Δ*f*) with strain (Δ*l*/*l_0_*), is fitted well to Δ*f* = 0.03Δ*l/l_0_*, where *l_0_* and Δ*l* represent the height of the original (2.5 mm) and compressed rubber, respectively (Figure 6p). Moreover, other NCP samples also present satisfactory sensitivity (Figure S19, Supporting Information). Compared to traditional ones, the EM sensor is more competitive in wireless and remote sensing and quick information feedback. Furthermore, the synergistic application of anti‐EM pollution and strain sensors will provide a new idea for health monitoring in the future.

## 3 Conclusion

We developed a novel approach to fabricate flexible electrothermal microwave absorbers for application in extreme service environments. The highly conductive Ni/CMT with a hierarchical hollow structure is uniformly dispersed within the flexible PTFE matrix to create a homogenous NCP film. Leveraging the remarkable polarization loss capacity of Ni/CMT and the fast heat dissipation channel, the resulting flexible NCP film showcases excellent Joule heating effect and MA performance. Particularly, under the action of 3 wt.% Ni/CMT, the NCP film realizes the low‐frequency MA with RL_min_ of 59.12 dB at 5.92 GHz. Under the action of 7 wt.% Ni/CMT, the NCP film exhibits broad MA with a bandwidth of 6.40 GHz at only 1.80 mm thickness. Additionally, CST simulation results further confirm the remarkable RCS reduction capacity, indicating a promising potential for MA applications. Basing on their unique EM features, a strain sensor device was designed and constructed. In summary, the combination of hierarchical Ni/CMT with flexible PTFE introduced in this study may offer significant alternatives for the development of multifunctional microwave absorbers. Accordingly, it is envisioned that these accomplishments may open a door for the designing and preparing of advanced MAMs for aerospace, aircraft, flexible device, and conducting composite fields.

## Conflict of Interest

The authors declare no conflict of interest.

## Supporting information

Supporting Information

## Data Availability

The data that support the findings of this study are available from the corresponding author upon reasonable request.

## References

[1] A. Iqbal , F. Shahzad , K. Hantanasirisakul , M. K. Kim , J. Kwon , J. Hong , H. Kim , D. Kim , Y. Gogotsi , C. M. Koo , Science 2020, 369, 446. 32703878 10.1126/science.aba7977

[2] X. Zhang , J. Grajal , J. L. Vazquez‐Roy , U. Radhakrishna , X. Wang , W. Chern , L. Zhou , Y. Lin , P. C. Shen , X. Ji , X. Ling , A. Zubair , Y. Zhang , H. Wang , M. Dubey , J. Kong , M. Dresselhaus , T. Palacios , Nature 2019, 566, 368. 30692651 10.1038/s41586-019-0892-1

[3] L. Feng , Y. L. Xu , W. S. Fegadolli , M. H. Lu , J. E. Oliveira , V. R. Almeida , Y. F. Chen , A. Scherer , Nat. Mater. 2013, 12, 108. 23178268 10.1038/nmat3495

[4] L. Liu , H. Deng , X. Tang , Y. Lu , J. Zhou , X. Wang , Y. Zhao , B. Huang , Y. Shi , Proc. Natl. Acad. Sci. USA 2021, 118, e2105838118. 34330835 10.1073/pnas.2105838118 PMC8346830

[5] B. Wen , M. Cao , M. Lu , W. Cao , H. Shi , J. Liu , X. Wang , H. Jin , X. Fang , W. Wang , Adv. Mater. 2014, 26, 3484. 24648151 10.1002/adma.201400108

[6] L. Huang , Y. Duan , Y. Shi , X. Ma , H. Pang , Q. Zeng , R. Che , Adv. Opt. Mater. 2022, 10, 2200249.

[7] Y. Guo , K. Ruan , G. Wang , J. Gu , Sci. Bull. 2023, 65, 1195. 10.1016/j.scib.2023.04.036 37179235

[8] X. X. Wang , W. Q. Cao , M. S. Cao , J. Yuan , Adv. Mater. 2020, 32, 2002112. 10.1002/adma.202002112 32686195

[9] G. Wang , Z. Gao , S. Tang , C. Chen , F. Duan , S. Zhao , S. Lin , Y. Feng , L. Zhou , Y. Qin , ACS Nano 2012, 6, 11009. 23171130 10.1021/nn304630h

[10] C. Liang , H. Qiu , P. Song , X. Shi , J. Kong , J. Gu , Sci. Bull. 2020, 65, 616. 10.1016/j.scib.2020.02.009 36659130

[11] M. Zhou , X. Xu , G. Wan , P. Mou , S. Teng , G. Wang , Nano Res. 2022, 15, 8677.

[12] L. Wu , X. Liu , G. Wan , X. Peng , Z. He , S. Shi , G. Wang , Chem. Eng. J. 2022, 448, 137600.

[13] Y. Zhang , L. Li , C. Du , G. Wan , Q. Wei , X. Zhou , Y. Su , Y. Xu , G. Wang , J. Mater. Sci. Technol. 2023, 151, 109.

[14] L. Liang , Q. Li , X. Yan , Y. Feng , Y. Wang , H. B. Zhang , X. Zhou , C. Liu , C. Shen , X. Xie , ACS Nano 2021, 15, 6622. 33780231 10.1021/acsnano.0c09982

[15] Y. Qiu , H. Yang , Y. Cheng , Y. Lin , Compos. Part A Appl. Sci. Manuf. 2021, 154, 106772.

[16] X. Liu , G. Wan , L. Wu , J. Liu , S. Shi , Q. Wei , G. Wang , Chem. Eng. J. 2023, 457, 141275.

[17] Q. Wei , L. Li , Z. Deng , G. Wan , Y. Zhang , C. Du , Y. Su , G. Wang , Small 2023, 2302082. 10.1002/smll.202302082 37105765

[18] L. Feng , P. Wei , Q. Song , J. Zhang , Q. Fu , X. Jia , J. Yang , D. Shao , Y. Li , S. Wang , X. Qiang , H. Song , ACS Nano 2022, 16, 17049. 36173441 10.1021/acsnano.2c07187

[19] P. He , W. Ma , J. Xu , Y. Wang , Z. K. Cui , J. Wei , P. Zuo , X. Liu , Q. Zhuang , Small 2023, 2302961.

[20] L. Yao , Y. Wang , J. Zhao , Y. Zhu , M. Cao , Small 2023, 19, e2208101. 36932880 10.1002/smll.202208101

[21] P. Liu , Y. Wang , G. Zhang , Y. Huang , R. Zhang , X. Liu , X. Zhang , R. Che , Adv. Funct. Mater. 2022, 32, 2202588.

[22] Y. Q. Wang , H. B. Zhao , J. B. Cheng , B. W. Liu , Q. Fu , Y. Z. Wang , Nano‐Micro Lett. 2022, 14, 76. 10.1007/s40820-022-00817-5 PMC8938554 35312846

[23] C. Cao , D. D. Ma , J. Jia , Q. Xu , X. T. Wu , Q. L. Zhu , Adv. Mater. 2021, 33, 2008631. 10.1002/adma.202008631 33988264

[24] F. Ran , X. Xu , D. Pan , Y. Liu , Y. Bai , L. Shao , Nano‐Micro Lett. 2020, 12, 46. 10.1007/s40820-020-0382-x PMC7770780 34138240

[25] G. Hai , Z. Tao , H. Gao , J. Zhao , G. Wang , Nano Energy 2021, 79, 105418.

[26] J. Yang , C. Yu , C. Hu , M. Wang , S. Li , H. Huang , K. Bustillo , X. Han , C. Zhao , W. Guo , Adv. Funct. Mater. 2018, 28, 1803272.

[27] Y. Hou , Z. Sheng , C. Fu , J. Kong , X. Zhang , Nat. Commun. 2022, 13, 1227. 35264594 10.1038/s41467-022-28906-4 PMC8907192

[28] Y. Zhang , K. Ruan , K. Zhou , J. Gu , Adv. Mater. 2023, 35, 2211642. 10.1002/adma.202211642 36703618

[29] B. Zhao , Y. Du , H. Lv , Z. Yan , H. Jian , G. Chen , Y. Wu , B. Fan , J. Zhang , L. Wu , D. W. Zhang , R. Che , Adv. Funct. Mater. 2023, 2302172.

[30] J. Cheng , H. Zhang , H. Wang , Z. Huang , H. Raza , C. Hou , G. Zheng , D. Zhang , Q. Zheng , R. Che , Adv. Funct. Mater. 2022, 32, 2201129.

[31] Y. Zhu , J. Zhang , Q. Qian , Y. Li , Z. Li , Y. Liu , C. Xiao , G. Zhang , Y. Xie , Angew. Chem., Int. Ed. 2022, 61, e202113082. 10.1002/anie.202113082 34669234

[32] R. Yang , J. Yuan , C. Yu , K. Yan , Y. Fu , H. Xie , J. Chen , P. K. Chu , X. Wu , J. Alloys Compd. 2020, 816, 152519.

[33] B. Zhao , X. Guo , W. Zhao , J. Deng , B. Fan , G. Shao , Z. Bai , R. Zhang , Nano Res. 2016, 10, 331.

[34] S. Singh , A. Kumar , S. Agarwal , D. Singh , J. Magn. Magn. Mater. 2020, 503, 166616.

[35] H. Wang , H. Guo , Y. Dai , D. Geng , Z. Han , D. Li , T. Yang , S. Ma , W. Liu , Z. Zhang , Appl. Phys. Lett. 2012, 101, 083116.

[36] L. Wang , M. Huang , X. Yu , W. You , J. Zhang , X. Liu , M. Wang , R. Che , Nano‐Micro Lett. 2020, 12, 150. 10.1007/s40820-020-00488-0 PMC7770844 34138180

[37] R. Yu , X. Wen , J. Liu , Y. Wang , X. Chen , K. Wenelska , E. Mijowska , T. Tang , Appl. Catal. B Environ. 2021, 298, 120544.

[38] X. Xu , G. Wang , G. Wan , S. Shi , C. Hao , Y. Tang , G. Wang , Chem. Eng. J. 2020, 382, 122980.

[39] P. Zhang , X. Zhang , B. Li , L. Xu , F. Dang , B. Li , Adv. Compos. Hybrid Mater. 2021, 4, 1292.

[40] L. Wu , S. Shi , G. Wang , P. Mou , X. Liu , J. Liu , L. Li , C. Du , Adv. Funct. Mater. 2022, 32, 2209898.

[41] P. Mou , G. Wan , L. Wu , D. Liu , G. Wang , J. Mater. Chem. A 2023, 11, 4345.

[42] D. Liu , P. Mou , Q. Wei , Y. Xu , G. Wan , G. Wang , Carbon 2023, 204, 7.

[43] M. Yuan , B. Zhao , C. Yang , K. Pei , L. Wang , R. Zhang , W. You , X. Liu , X. Zhang , R. Che , Adv. Funct. Mater. 2022, 32, 2203161.

[44] W. Gu , J. Sheng , Q. Huang , G. Wang , J. Chen , G. Ji , Nano‐Micro Lett. 2021, 13, 102. 10.1007/s40820-021-00635-1 PMC8021664 34138342

[45] H. Li , Y. Fu , D. Alhashmialameer , H. K. Thabet , P. Zhang , C. Wang , K. Zhu , M. Huang , Z. Guo , F. Dang , Adv. Compos. Hybrid Mater. 2022, 5, 2631.

[46] T. T. Liu , Y. H. Zhu , J. C. Shu , M. Zhang , M. S. Cao , Mater. Today Phys. 2023, 31, 100988.

[47] M. Zhang , C. Han , W. Q. Cao , M. S. Cao , H. J. Yang , J. Yuan , Nano‐Micro Lett. 2020, 13, 27. 10.1007/s40820-020-00552-9 PMC8187527 34138252

[48] M. S. Cao , X. X. Wang , M. Zhang , W. Q. Cao , X. Y. Fang , J. Yuan , Adv. Mater. 2020, 32, 1907156.

