# Supplementary material for: Ni‐Carbon Microtube/Polytetrafluoroethylene as Flexible Electrothermal Microwave Absorbers
Source: Adv Sci (Weinh). 2023 Sep 18;10(31):2304218. doi: 10.1002/advs.202304218 (PMC10625052; doi:10.1002/advs.202304218)
Supplement: Supplementary file 1 — Supporting Information [file ADVS-10-2304218-s001.pdf]

ADVANCED SCIENCE
Open Access

Supporting Information

for *Adv. Sci.*, DOI 10.1002/advs.202304218

Ni-Carbon Microtube/Polytetrafluoroethylene as Flexible Electrothermal Microwave Absorbers

Lihong Wu, Guizhen Wang, Shaohua Shi, Xiao Liu, Jun Liu, Jinchuan Zhao and Guilong Wang**

Supporting Information

Ni-Carbon Microtube/Polytetrafluoroethylene as Flexible Electrothermal Microwave Absorbers

Lihong Wu, Guizhen Wang, Shaohua Shi, Xiao Liu, Jun Liu, Jinchuan Zhao, and Guilong Wang**

L. Wu, G. Wang, S. Shi, X. Liu, J. Liu, J. Zhao
State Key Laboratory of Marine Resource Utilization in South China Sea, School of Material Science and Engineering, Hainan University, Haikou, Hainan 570228, China
Email: wangguizhen0@hotmail.com

G. Wang
Key Laboratory for Liquid-Solid Structural Evolution and Processing of Materials (Ministry of Education), Shandong University, Jinan, Shandong 250061, China
Email: guilong@sdu.edu.cn

Materials

The kapok fiber (KF) was obtained from Hainan. Dimethyl formamide (DMF), methanol, ethanol, nickel nitrate hexahydrate ($Ni(NO_3)_2{\cdot}6H_2O$), and trimesic acid (H_3BTC) were purchased from Macklin Biochemical Technology Co., Ltd. PTFE aqueous dispersion (60 wt.%) was purchased from Daikin Fluorochemicals Co. Ltd., China.

Preparation of CMT

Typically, the KF was placed in a tube furnace and thermally treated at 800 ℃ for 2h under a nitrogen atmosphere with a heating rate of 5 ℃ min^{-1}. The carbonized KF was named CMT.

Synthesis of Ni-MOF/CMT Composites

150 mg of CMT was dispersed in the 60 mL of DMF containing 100 mg of H_3BTC under magnetic agitation, followed by the addition of 870 mg of $Ni(NO_3)_2{\cdot}6H_2O$. The mixture was transferred into a 100 mL of Teflon-lined stainless-steel autoclave and kept at 150 °C for 12 h. The products were soaked in methanol for 48 h to remove the solvent and unreacted ligand. Subsequently, the composites were centrifuged and washed with water and ethanol. Finally, the products dried in a vacuum at 60 °C for 24 h. The final products were decorated as Ni-MOF/CMF-*x* (*x* = 50, 100, 150), where *x* represents the amount of CMT. For comparison, the pure Ni-MOF was prepared under the same experimental procedure.

Synthesis of Ni/CMT Composites

Ni-MOF/CMT-150 was heated at 450 ℃ at a ramp rate of 2 ℃ min^{-1} under a nitrogen atmosphere. After keeping the temperature for 2 h, the system was cooled to room temperature, and Ni/CMT was obtained.

Synthesis of NCP Films

The NCP films were prepared through the simple mechanical mixing of PTFE and Ni/CMT. In a typical process, the Ni/CMT was dispersed in PTFE. Then, a suitable amount of ethanol was added to the mixed solution after stirring for half an hour at room temperature. Mechanical stirring and the addition of ethanol to the mixture of Ni/CMT and PTFE both resulted in rapid demulsification. During the process of demulsification, extended PTFE fibers were formed. These fibrillated networks wrapped around the uniformly dispersed Ni/CMT to obtain homogeneous textured Ni/CMT/PTFE films. Finally, the NCP with a mass ratio of Ni/CMT and PTFE of 1 wt.%, 3 wt.%, 5 wt.%, and 7 wt.% were designated as NCP -1, NCP-3, NCP-5, and NCP-7.

Apparatus

X-ray photoelectron spectroscopy (XPS) data were recorded by AXIS SUPRA. The phase composition and crystal structure were characterized by X-ray diffraction (XRD; Smart Lab II). Scanning electron microscopy (SEM), and energy dispersive spectroscopy (EDS) mappings were performed by using the Thermo Scientific Verios G4 UC microscope. Transmission electron microscopy (TEM), and EDS mapping of samples were collected by using the transmission electron microscopy system (Thermo Scientific, Talos F200X G2). Uniaxial tensile measurements were performed by an electronic universal testing machine (ETM series, WANCE) at room temperature with a strain rate of 50 mm min^{-1}. The room temperature thermal diffusivity (α) values were obtained by a thermal constant analyzer (Netzsch LFA 467 Hyper Flash). The thermal conductivity (λ) of samples was according to the equation: $\lambda = \rho \times Cp \times \alpha$, where ρ is density, *Cp* specific heat. The temperature of the NCP was monitored with an infrared thermal imager (FLIR E6). Contact angles of the samples were measured by an optical contact angle system (Attension theta flex). The I−V curve of the composites was measured by an electrochemical workstation (CH Instruments, CHI660D). The resistances (R) of composites were collected by a digital multimeter (Tektronix DMM6500). Electrical conductivity was calculated using the formulated: $\sigma = L/SR$ (L, S, and R are the length, cross-sectional area, and resistance of the samples, respectively). The joule heating test was conducted by connecting film samples to a power supply (UTP–1310, UNI–T Co., China) at constant voltages. Infrared thermography (FLIR, E5–XT) was used to record the temperature change of the composites during the heating process. The limiting oxygen index (LOI) values were analyzed on an HC-2C oxygen index meter (Jingning Analysis Instrument Company).

Microwave Absorption Properties Test

To obtain the microwave absorption performance of as-prepared composites, the EM parameters were performed in the frequency range of 2–18 GHz using a network analyzer (Ceyear, 3672B-S). The obtained Ni-MOF/CMT were mixed with paraffin at a mass ratio of 10 wt.%, followed by shaping into sizes of an outer diameter of 7.00 mm and an inner diameter of 3.04 mm (2.00 mm in thickness). The NCP composites were cut into circular coils of 7.00 mm outer diameter and 3.04 mm inner diameter.

Simulation

A numerical model was carried out using COMSOL Multiphysics with the RF module to investigate the current density of composites. The simulation models of the samples were obtained with a periodic unit model. To investigate the influence on the electric field intensity of NCP, corresponding simulation models were built. The incident microwave with a power of 1 W at the frequency of 15 GHz was excited from the port I and injected into the MA material along the z-axis (the direction of electric field polarization and magnetic field polarization was along the x-axis and along the y-axis, respectively). Perfectly matched layers (PML) at the top and bottom of the computational domain are applied to absorb the reflected and transmitted microwaves, respectively.

A radar cross-section (RCS) simulation was carried out to evaluate the actual far-field condition MA properties in real situations, which is simulated by CST Studio Suite 2020. The frequency of 8 GHz (NCP-1), 6 GHz (NCP-3), 11 GHz (NCP-5), and 15 GHz (NCP-7) are selected. According to the widely accepted metal back model, the simulation model of the specimens was established as a square (200 mm × 200 mm) with dual layers. In detail, the bottom set as 0.5 mm is the perfect conductive layer (PEC), and the upper set as 5.0 mm, 5.0 mm, 2.6 mm, and 1.8 mm signifies the absorbing layer for NCP-1, NCP-3, NCP-5, and NCP-7, respectively. The PEC and the absorbing model plate were placed on the X–O–Y plane and linear polarized plane EM waves incident from the positive direction of the Z-axis to the negative direction of the Z-axis. Simultaneously, the direction of electric polarization propagation is along the X-axis. Open (add space) boundary conditions were employed in the x, y, and z directions. Generally, the scattering directions of RCS value can be determined by theta and phi in spherical coordinates, which can be described below:

$$\sigma = 10\log\left((4\pi S/\lambda^2)|E_S/E_i|\right)^2$$

Here, S, λ, Es, and Ei are the area of the target object simulation model, the wavelength of EM wave, the electric field intensity of scattered wave, and the incident wave, respectively.

An EM sensor was investigated by CST Microwave Studio. The boundary conditions were applied with the electric field along *x* direction and the magnetic field along *y* direction. The NCP composites were applied as a pyramid pattern and a substrate. The middle layer is rubber with 2 mm, which is high elasticity and wave-transparent material (ε = 3). The top layer is copper with 100 μm thickness ($\sigma = 5.96\times10^7$ S m^{-1}). The *l* is applied the between pyramid pattern and substrate. The working principle of the sensor is that when the microwave of 2-18 GHz is incident on the

sensor, the dielectric loss and coupled effect occur to dissipate the energy of the microwave, with the appearance of a strong absorption peak. At that time, the absorption peak can be as an output signal, which is sensitive to pressure (characterized by the distance of a pyramid pattern and a substrate).

Equations

$$Z_{in} = Z_0\sqrt{\mu_r/\varepsilon_r}\,tanh[j(2\pi f d/c)\sqrt{\mu_r\varepsilon_r}] \quad \text{(S1)}$$

$$RL = 20\,lg|(Z_{in} - Z_0)/(Z_{in} + Z_0)| \quad \text{(S2)}$$

Where Z_{in} is the input impedance of the absorber, Z_0 is the impedance of free space, μ_r is the relative complex permeability ($\mu_r = \mu' - i\mu''$), ε_r is the complex permittivity ($\varepsilon_r = \varepsilon' - i\varepsilon''$), d is the thickness of absorber, c and f are velocity and frequency of light, respectively.

In general, the dielectric loss can be described by Debye's theory:

$$\varepsilon' = \varepsilon_\infty + \frac{\varepsilon_s - \varepsilon_\infty}{1+(\omega\tau_0)^2} \quad \text{(S3)}$$

$$\varepsilon'' = \frac{\omega\tau_0(\varepsilon_s - \varepsilon_\infty)}{1+(\omega\tau_0)^2} + \frac{\sigma}{\omega\varepsilon_0} \quad \text{(S4)}$$

where, ε_∞ is the relative dielectric permittivity in the high-frequency limit, τ is the polarization relaxation time, ω is the angular frequency, ε_s is the static permittivity, ε_0 is the dielectric constant of vacuum, σ is the electrical conductivity.

The attenuation constant (α) represents the attenuation capacity of EM wave, which is usually expressed as follows

$$\alpha = \frac{\sqrt{2}\pi f}{c} \times \sqrt{(\mu''\varepsilon'' - \mu'\varepsilon') + \sqrt{(\mu''\varepsilon'' - \mu'\varepsilon')^2 + (\mu''\varepsilon' + \mu'\varepsilon'')^2}} \quad \text{(S5)}$$

For the relaxation losses, the Cole-Cole plots are used to analyze the polarization mechanism, according to the following equation:

$$\left(\varepsilon' - \frac{\varepsilon_s + \varepsilon_\infty}{2}\right)^2 + (\varepsilon'')^2 = \left(\frac{\varepsilon_s + \varepsilon_\infty}{2}\right)^2 \quad \text{(S6)}$$

The quarter-wavelength (λ/4) theory can be used to explain the relationship between matching thickness (t_m) and absorption peak frequency (f_m), which can be expressed as follows:

$$t_m = \frac{n\lambda}{4} = \frac{nc}{4f_m\sqrt{|\varepsilon_r\mu_r|}}\ (n = 1, 3,5 \ldots.) \quad \text{(S7)}$$

Figures

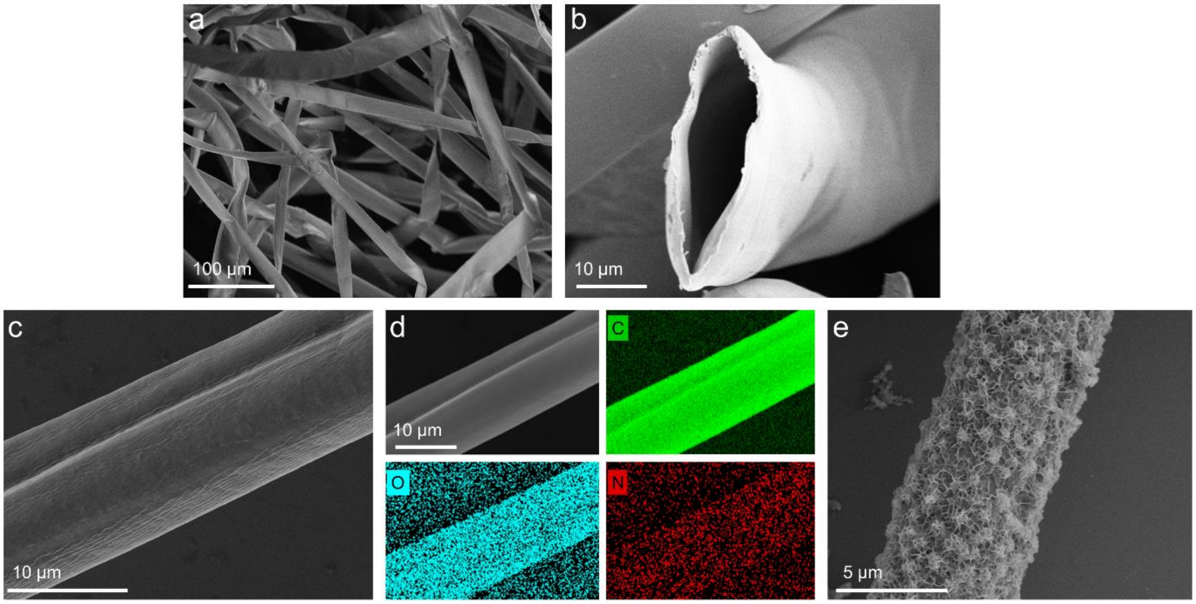

Figure S1. SEM images of (a,b) KF and (c) CMT. EDS elemental mappings of CMT. (e) SEM image of Ni-MOF/CMT-150.

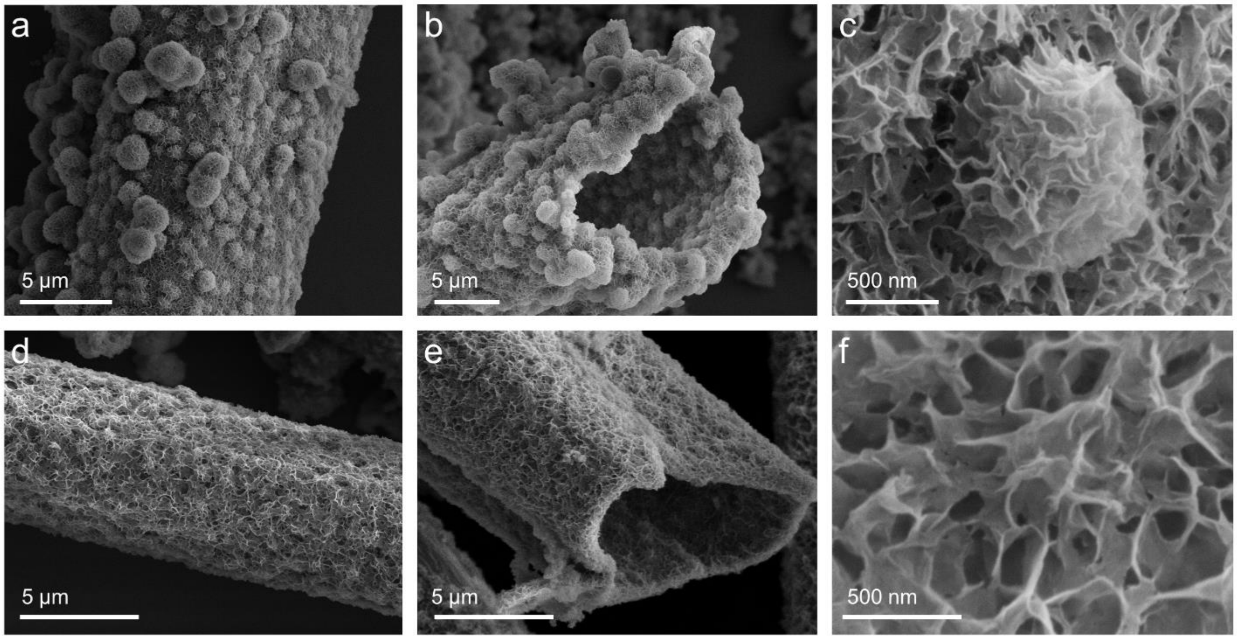

Figure S2. SEM images of (a–c) Ni-MOF/CMT-50 and (d–f) Ni-MOF/CMT-100.

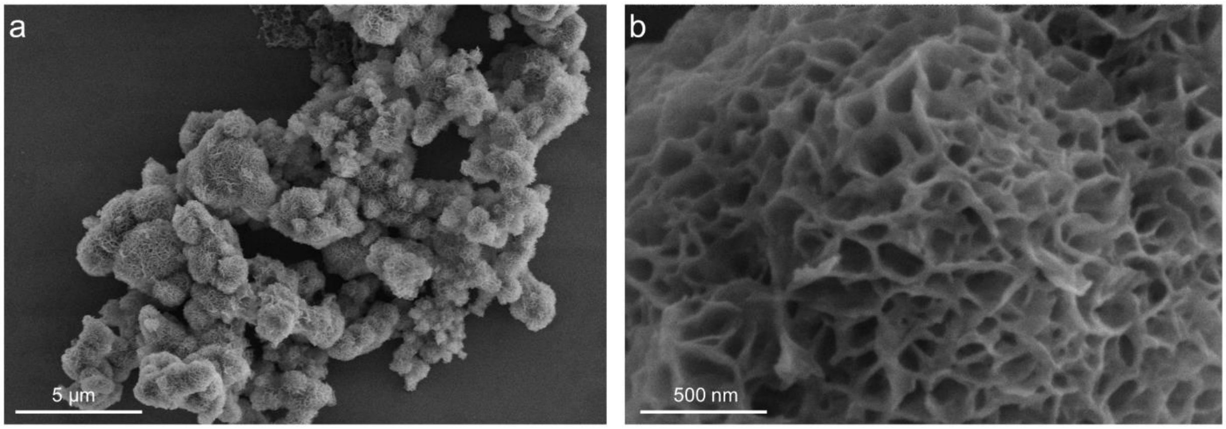

Figure S3. SEM images of Ni-MOF.

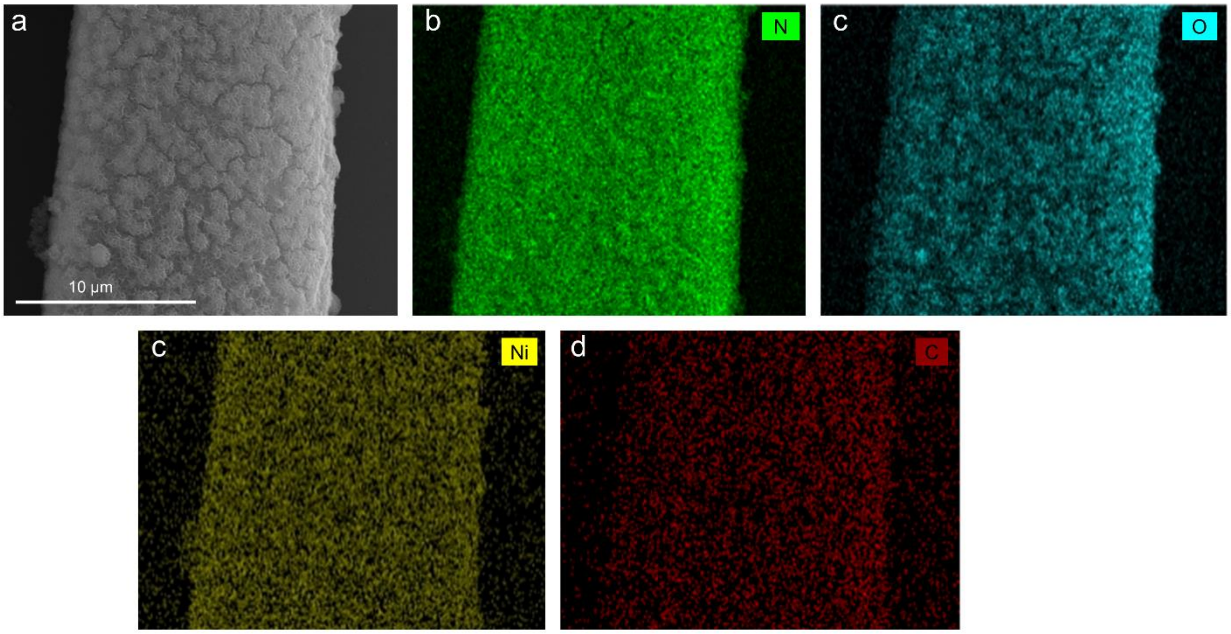

Figure S4. EDS elemental mappings of Ni-MOF/CMT-100.

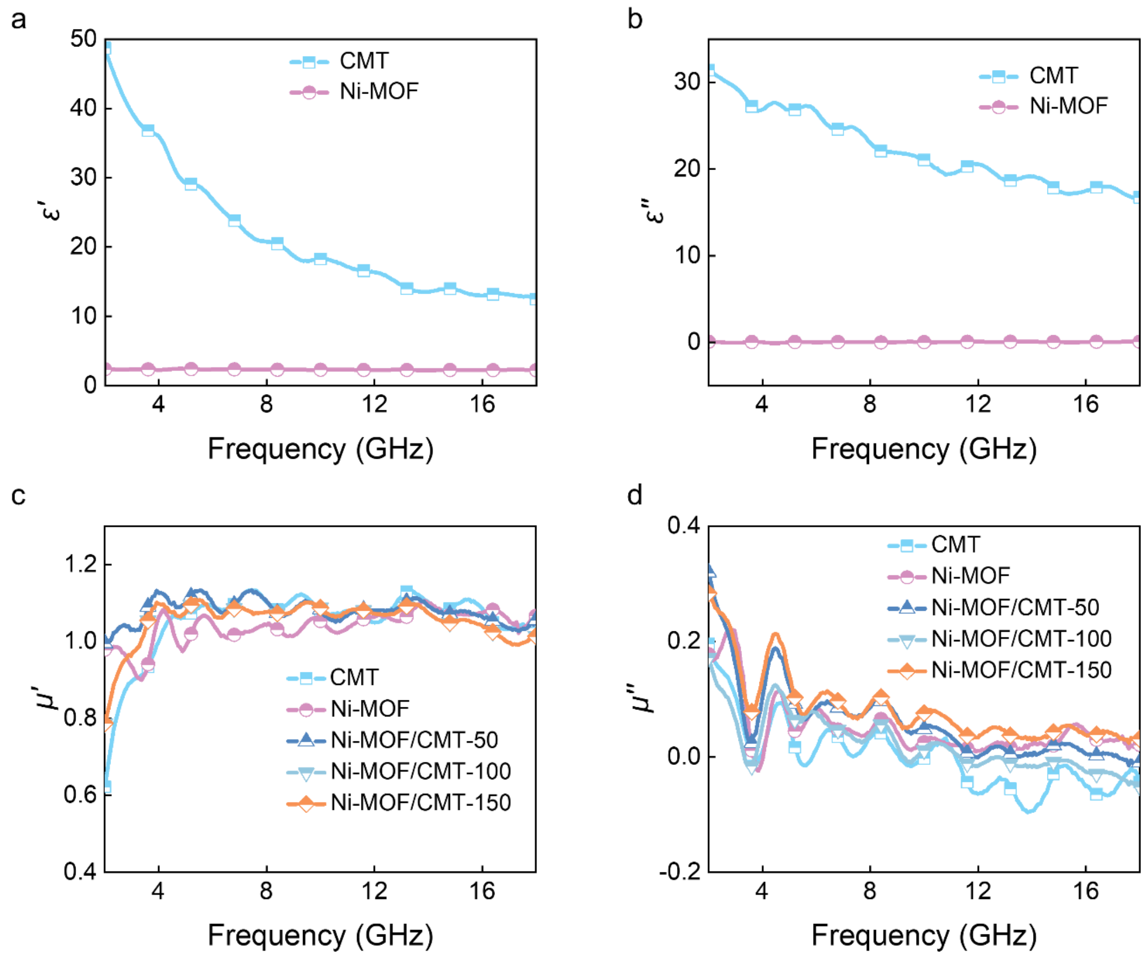

Figure S5. (a) ε' and (b) ε'' of Ni-MOF and CMT. (c) μ' and (d) μ'' values of as-prepared samples.

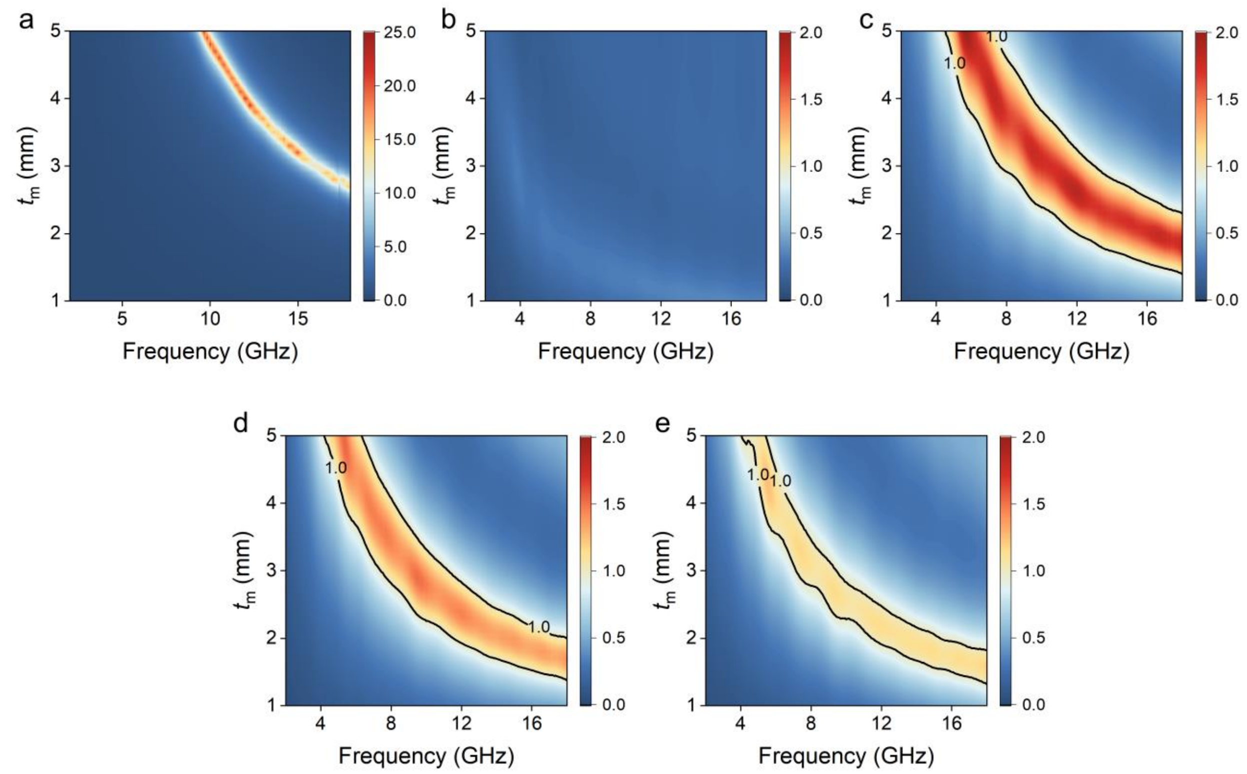

Figure S6. Impedance matching values of as-prepared samples. (a) Ni-MOF, (b) CMT, (c) Ni-MOF/CMT-50, (d) Ni-MOF/CMT-100, and (e) Ni-MOF/CMT-150.

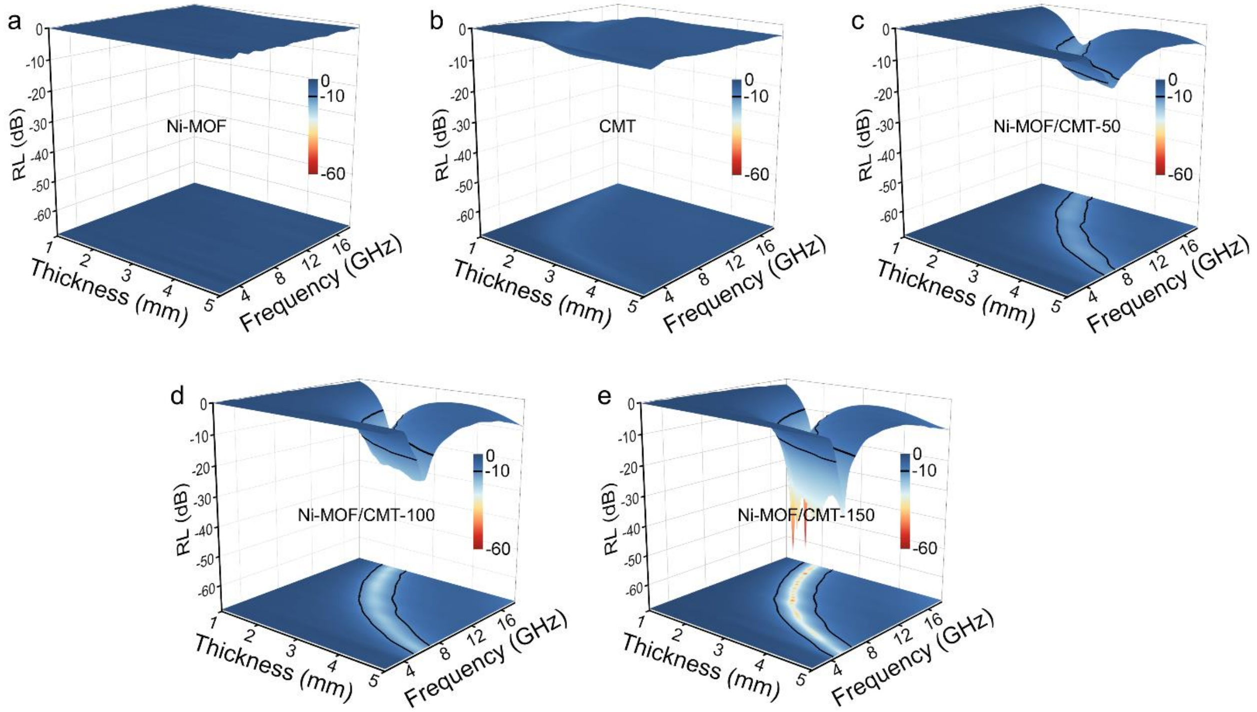

Figure S7. 3D RL values of as-prepared samples. (a) Ni-MOF, (b) CMT, (c) Ni-MOF/CMT-50, (d) Ni-MOF/CMT-100, and (e) Ni-MOF/CMT-150.

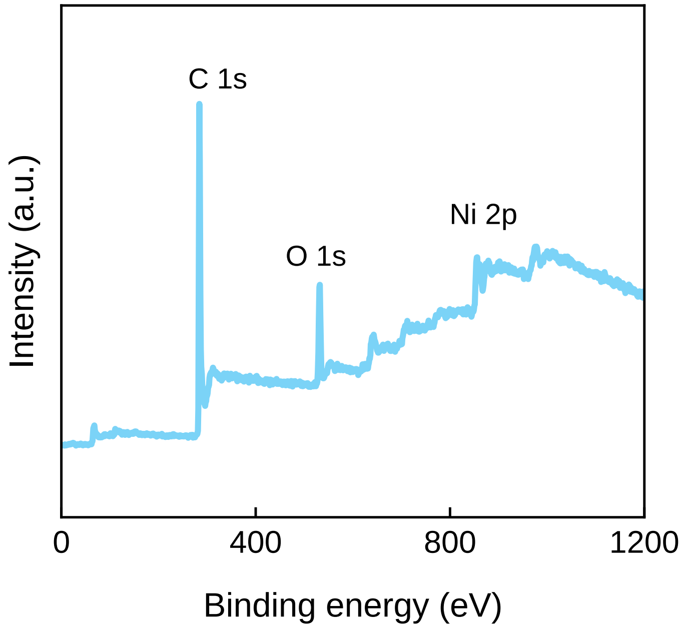

Figure S8. XPS spectrum of Ni/CMT.

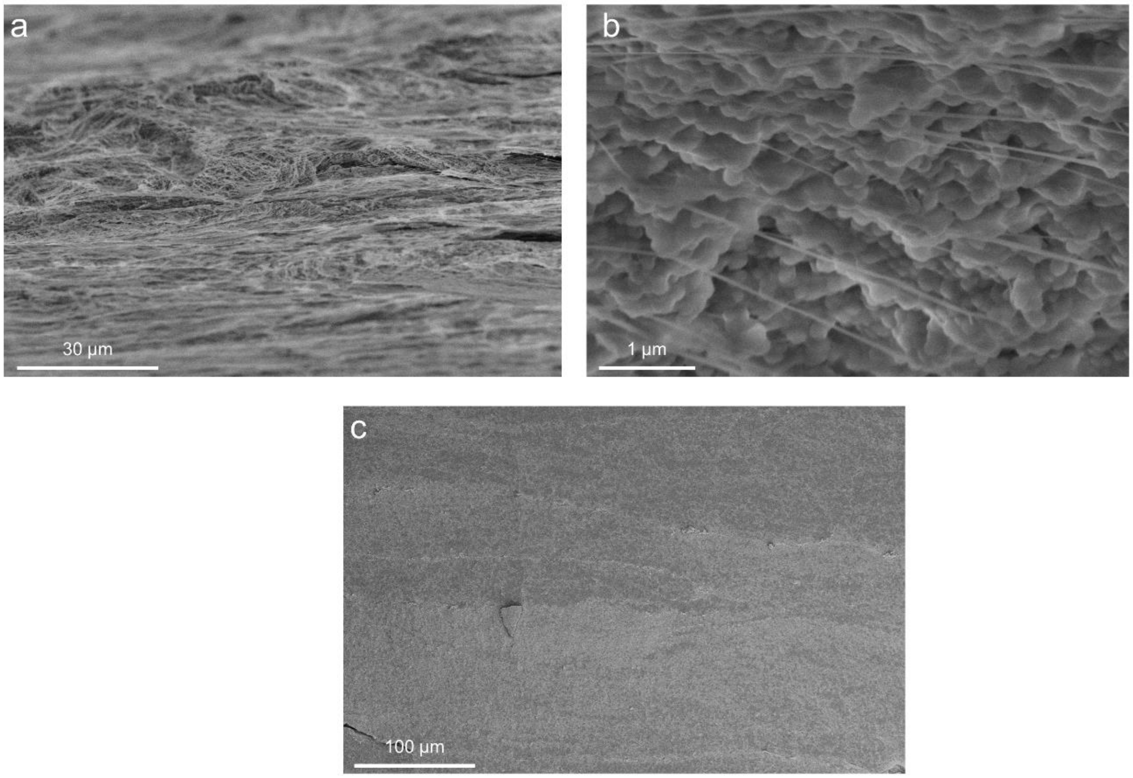

Figure S9. SEM images of (a,b) PTFE (NCP-0) and (c) surface-view image of the NCP film.

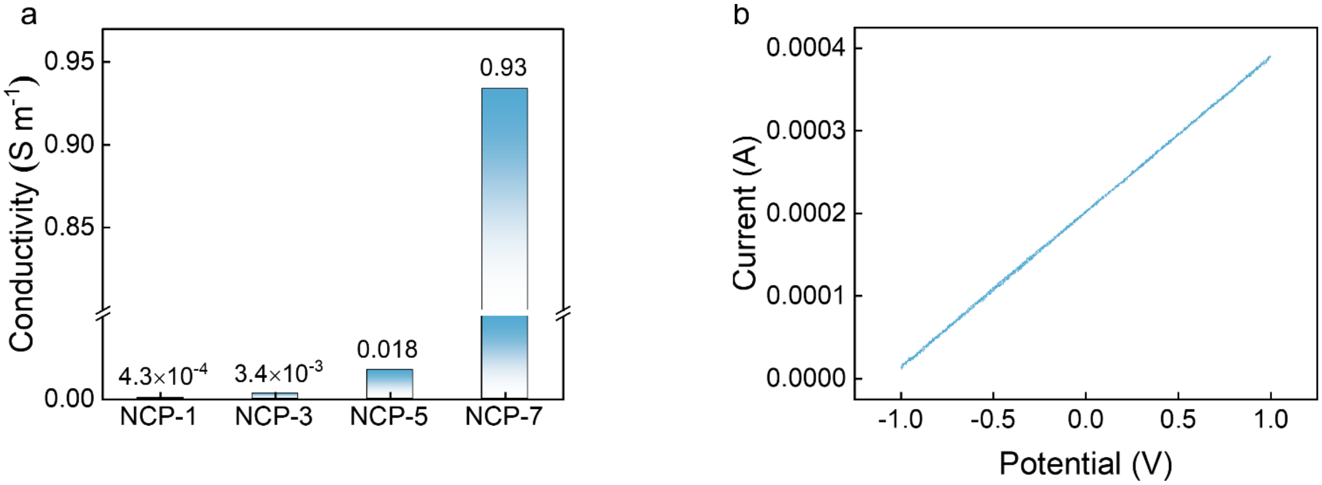

Figure S10. (a) Electrical conductivities of NCP films. (b) V–I linear curve NCP-7.

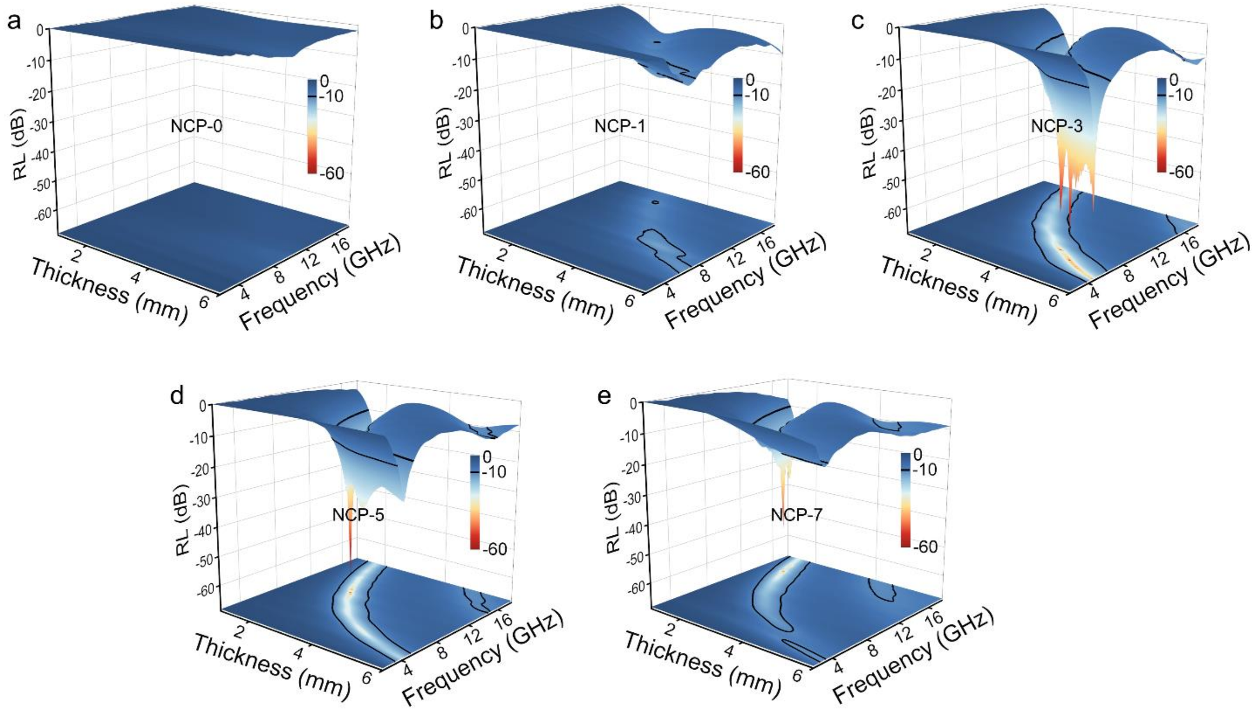

Figure S11. 3D RL values of NCP samples. (a) NCP-0, (b) NCP-1, (c) NCP-3, (d) NCP-5, and (e) NCP-7.

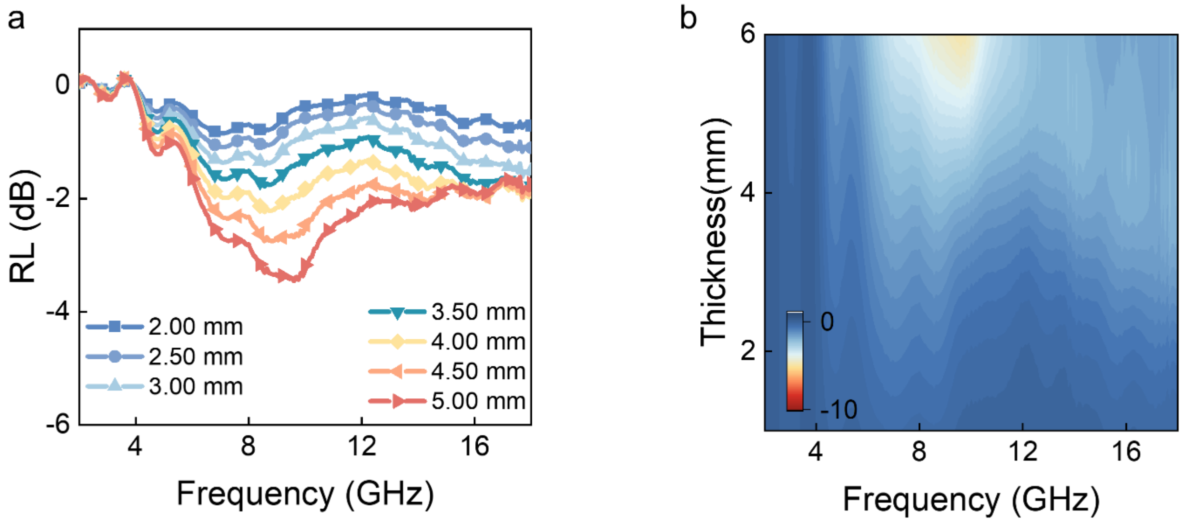

Figure S12. RL curves of NCP-0.

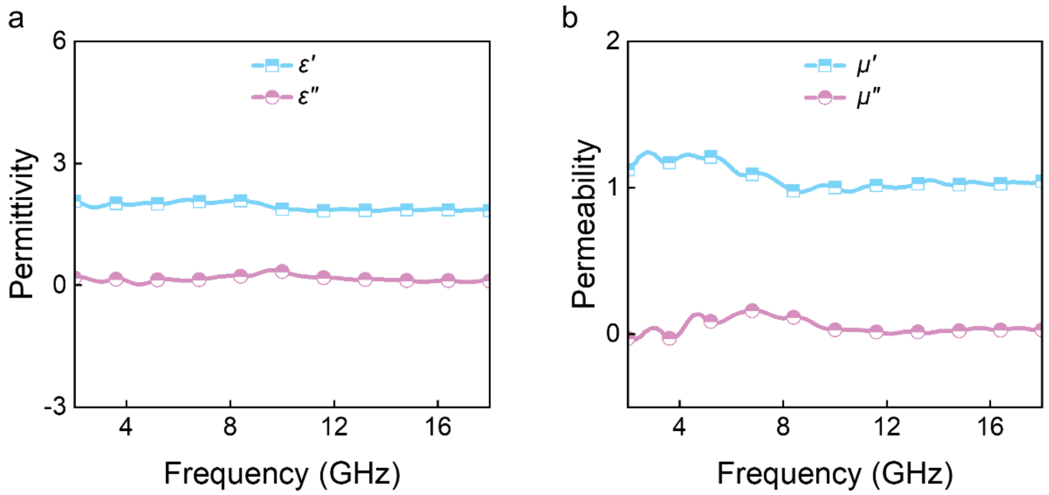

Figure S13. EM parameter of NCP-0. (a) Permittivity and (b) permeability.

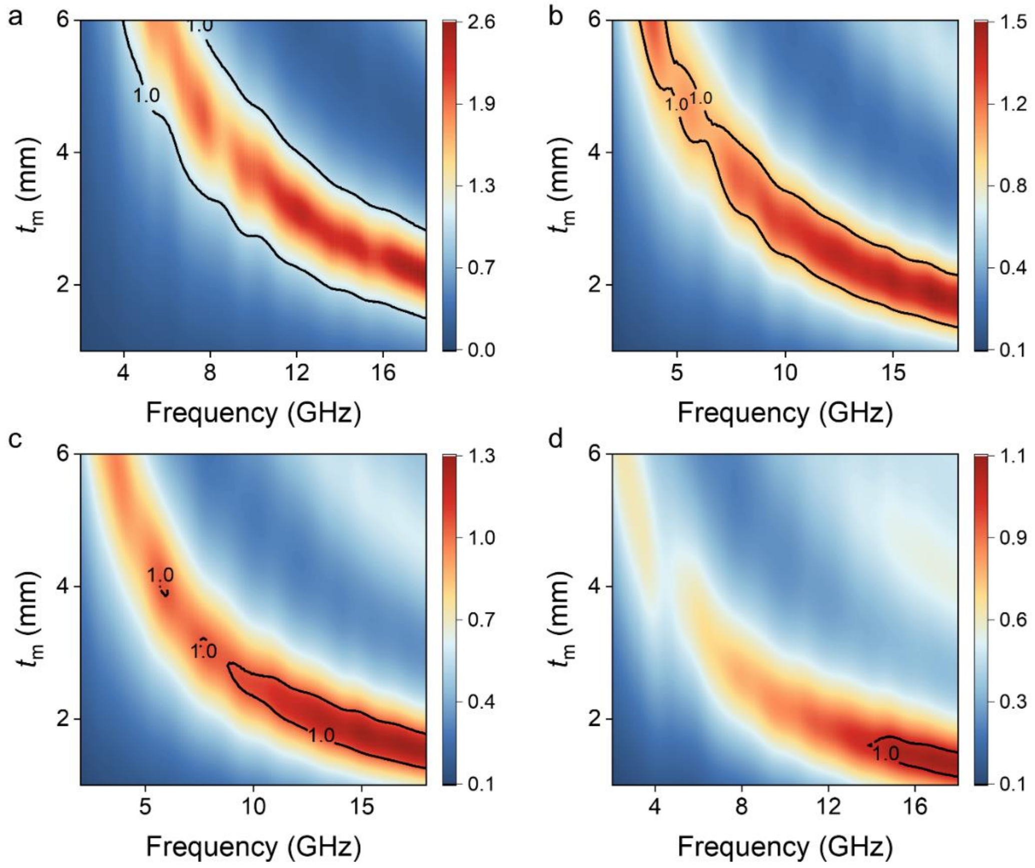

Figure S14. Impedance matching values of NCP films. (a) PCN-1, (b) PCN-3, (c) PCN-5, and (d) PCN-7.

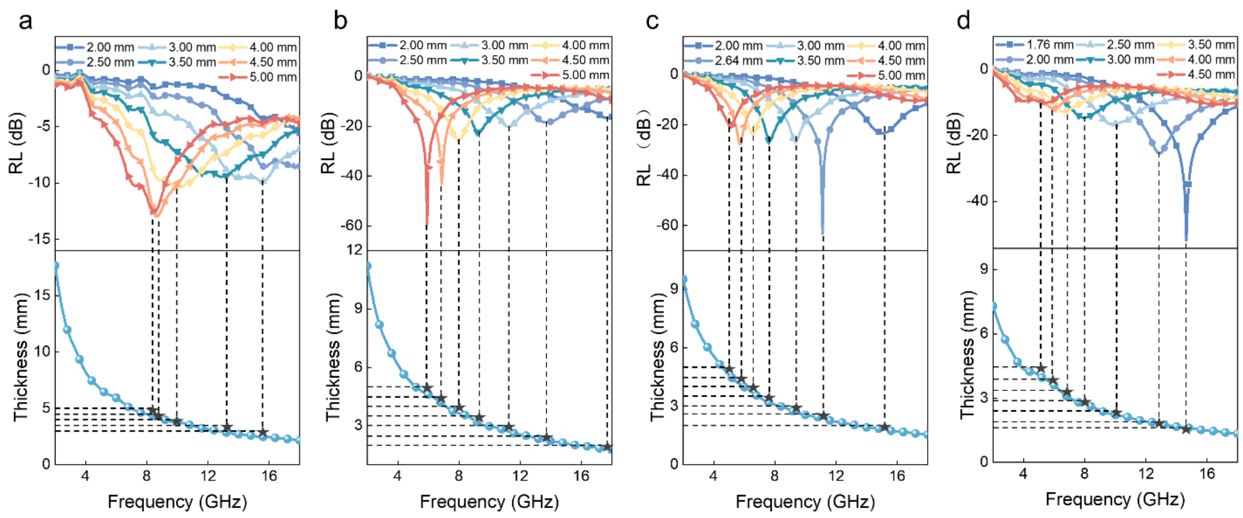

Figure S15. λ/4 matching theory. The RL dependence of t_m on f_m at $\lambda/4$ of (a) NCP-1, (b) NCP-3, (c) NCP-5, and (d) NCP-7.

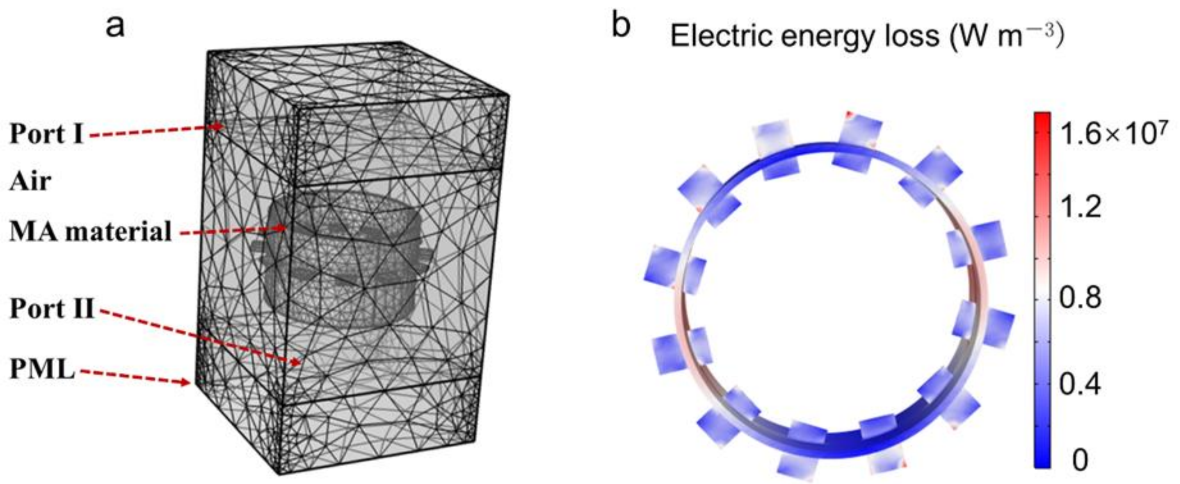

Figure S16. Modeling and simulation. (a) Modeling diagram and (b) electric energy loss distribution.

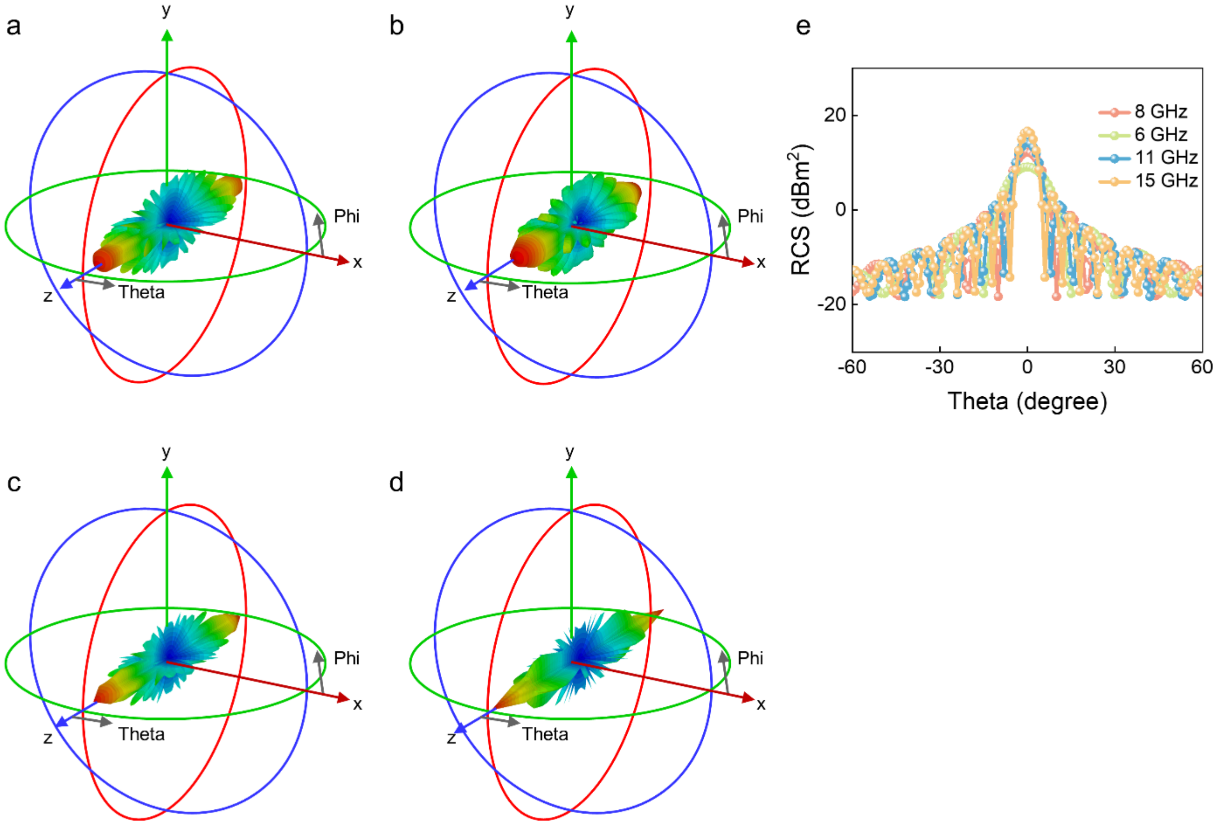

Figure S17. CST simulation results of PEC. 3D RCS diagram for the PEC substrate at (a) 8 GHz, (b) 6 GHz, (c) 11 GHz, and (d) 12 GHz. (e) RCS simulated curves under certain detecting angles.

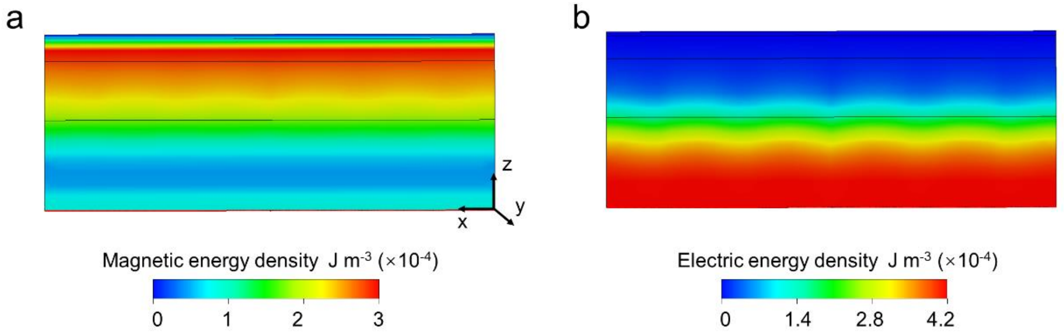

Figure S18. Energy density distributions. (a) Magnetic energy density distributions and (b) electric field density distributions of NCP-7 pattern at *l* of 0.3 mm (*f* = 9 GHz).

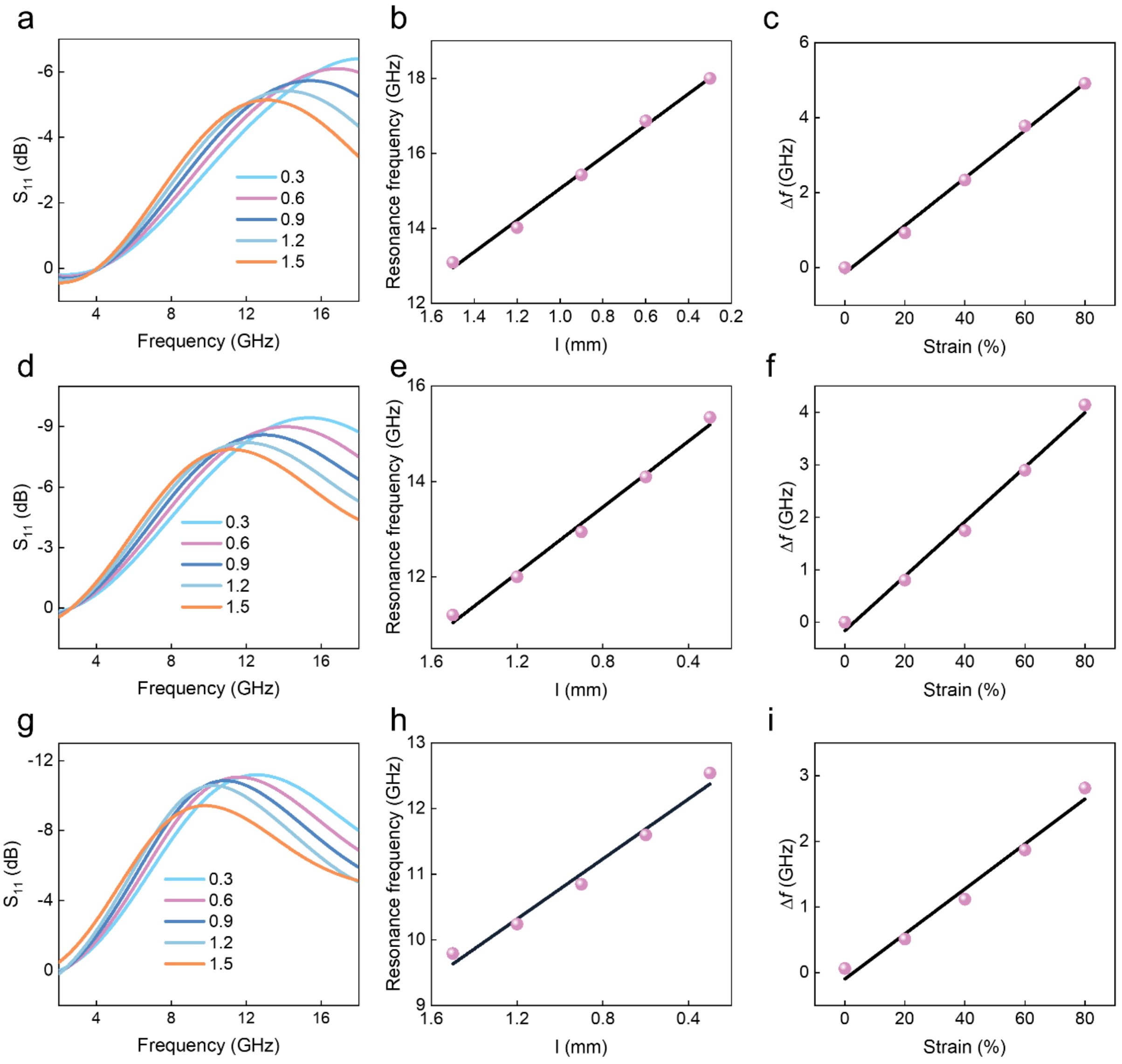

Figure S19. CST simulation pressure-driven EM sensor. Frequency-dependence S_{11} at $l = 0.3-1.5$ mm of (a) NCP-1, (d) NCP-3, (g) NCP-5. Resonance frequency response fitted at various l of (b) NCP-1, (e) NCP-3, (h) NCP-5. Fitted pressure-response curve of (c) NCP-1, (f) NCP-3, and (i) NCP-5.

Table S1. LOI tests of NCP samples.

Samples	LOI (%)
NCP-0	94.8
NCP-1	95.1
NCP-3	95.6
NCP-5	95.8
NCP-7	95.9
